# MmuPV1 infection of *Tmc6*/*Ever1* or *Tmc8*/*Ever2* deficient FVB mice as a model of βHPV in typical epidermodysplasia verruciformis

**DOI:** 10.1371/journal.ppat.1012837

**Published:** 2025-01-15

**Authors:** Margaret Wong, Hsin-Fang Tu, Ssu-Hsieh Tseng, Rebecca Mellinger-Pilgrim, Simon Best, Hua-Ling Tsai, Deyin Xing, Chien-fu Hung, Paul F. Lambert, Richard B. S. Roden

**Affiliations:** 1 Department of Pathology, Johns Hopkins University, Baltimore, Maryland, United States of America; 2 Department of Otolaryngology-Head and Neck Surgery, Johns Hopkins Medical Institution, Baltimore, Maryland, United States of America; 3 Oncology Biostatistics, Johns Hopkins University, Baltimore, Maryland, United States of America; 4 McArdle Laboratory for Cancer Research, University of Wisconsin-Madison, Madison, Wisconsin, United States of America; University of North Carolina at Chapel Hill, UNITED STATES OF AMERICA

## Abstract

Typical epidermodysplasia verruciformis (EV) is a rare, autosomal recessive disorder characterized by an unusual susceptibility to infection with specific skin-trophic types of human papillomavirus, principally *betapapillomaviruses*, and a propensity for developing malignant skin tumors in sun exposed regions. Its etiology reflects biallelic loss-of-function mutations in *TMC6* (*EVER1*), *TMC8* (*EVER2*) or *CIB1*. A TMC6-TMC8-CIB1 protein complex in the endoplasmic reticulum is hypothesized to be a restriction factor in keratinocytes for βHPV infection. However, the complex is also present in lymphocytes and its loss may compromise cellular immune control of βHPV infection. Indeed, certain primary immunodeficiencies, iatrogenic immunosuppression and AIDS are associated with the atypical form of EV. While well controlled in immunocompetent mice, murine papillomavirus MmuPV1 was first isolated from immunodeficient mice with florid skin warts, modeling atypical EV. To examine their potential as a model of typical EV, *Tmc6*^-/-^, *Tmc8*^-/-^ or wildtype FVB mice were challenged with MmuPV1. At day 16 post vaginal challenge with MmuPV1, the levels of viral transcripts were similar in *Tmc6*^-/-^ and *Tmc8*^-/-^ mice and wildtype FVB mice, arguing against Tmc6/8 acting as intracellular restriction factors. Thereafter, greater clearance of MmuPV1 by the wildtype that the *Tmc6*^-/-^ and *Tmc8*^-/-^ FVB mice was evident, supporting the hypothesis that typical EV reflects a subtle cellular immune deficit. Indeed, *Tmc6*^*-/-*^ or *Tmc8*^*-/-*^ mice exhibit partial CD8 T cell deficits and elevated Treg. While interferon-γ production and surface CD25 were similarly elevated in CD8 T cells upon in vitro stimulation with anti-CD3/CD28, the fraction of *Tmc6*^-/-^ or *Tmc8*^-/-^ CD8 T cells that were dividing was lower compared to wildtype. Typical EV patients exhibit normal control of most viral infections; *Tmc6*^-/-^, *Tmc8*^-/-^ and wildtype FVB mice similarly controlled vaccinia virus after skin challenge and induced neutralizing antibodies.

## Author summary

Typical epidermodysplasia verruciformis (EV) patients carry biallelic disabling mutations in *TMC6*, *TMC8* or *CIB1*, and suffer high rates of skin cancer in UV-exposed sites associated with human betapapillomavirus (βHPV)+ plane warts. βHPV infections are common, but asymptomatic in healthy individuals. Typical EV is not associated with enhanced susceptibility to other infectious agents, and is proposed to reflect a loss of keratinocyte-intrinsic immunity specific for βHPV. Atypical EV is driven by certain inherited T cell deficits, AIDS or immunosuppressive drugs. βHPV and mouse papillomavirus MmuPV1 each lack *E5*, utilize similar oncogenic pathways, and synergize with UV and immunosuppression to promote skin cancer. We show establishment of MmuPV1 infection is similar to wildtype FVB mice, but thereafter more persistent in *Tmc6*^*-/-*^ or *Tmc8*^*-/-*^ mice. *Tmc6*^*-/-*^ or *Tmc8*^*-/-*^ mice exhibit partial CD8 T cell deficits and elevated Treg, but normal control of vaccinia, implying typical EV actually reflects subtle T cell dysfunction.

## Introduction

The >200 known HPV genotypes are classified into distinct genera (α,β,γ,μ, etc) based upon their biological characteristics and sequence [1]. The 50 known cutaneous βHPVs are biologically and immunologically distinct from the high risk (hr) genital αHPVs that cause cervical and subsets of other anogenital cancers and oropharyngeal cancer [2]. While UV exposure is clearly the primary driver of cutaneous squamous cell carcinoma (CSCC), there is evidence for cutaneous βHPV infection as a co-carcinogen in certain high risk cohorts, and potentially even otherwise healthy but immunosenescent individuals [3]. The βHPVs are associated with CSCC in organ transplant recipients (OTR) and HIV+ patients, but the association with CSCC in aging but healthy individuals is less clear. βHPV replication, especially when elevated in the absence of effective immune control, may compromise repair of UV-induced DNA damage in the skin [3]. βHPV episomes and early gene expression can readily be detected in the precursor lesions, actinic keratosis, and in well-differentiated keratinizing SCC, but not in poorly differentiated CSCC. Thus, in contrast to the retention of oncogenic αHPV in 99% of cervical cancers, the continued presence of βHPV may not be required in poorly differentiated non-keratinizing SCC, but instead act as an impediment to its growth. A ‘hit-and-run’ mechanism has been proposed wherein the presence of βHPV is subject to negative selection in poorly differentiated non-keratinizing SCC once the UV-induced transforming mutations have been established because of ineffective DNA repair [3–6]. Establishing βHPV as a co-factor in the development of CSCC is especially challenging not only because the proposed mechanism is so distinct from direct hrHPV-driven cervical carcinogenesis, but also βHPV infection is so prevalent [7]. Indeed, its role remains controversial [8] as the landscape of driver mutations is similar in CSCC of immunosuppressed and competent patients [9], and βHPV may be a bystander infection or possibly even a symbiont that is protective against CSCC [10–12].

The association of CSCC with a certain βHPVs was initially described in the inherited syndrome epidermodysplasia verruciformis (EV) [13–15]. Typical EV is a rare autosomal recessive genetic disease associated with biallelic inactivating *TMC6*, *TMC8* (also called *EVER1* and *EVER2*, respectively) [16] or *CIB1* germline mutations [17]. While an extensive cutaneous burden of plane warts is characteristic of patients with EV, infections in the genital area have also been reported [18–20]. TMC6, TMC8 and CIB1 form a heterotrimeric protein complex in the endoplasmic reticulum of unknown function [21], but they have been proposed to govern keratinocyte-intrinsic immunity to βHPV [22]. Typical EV patients appear to have a generally intact immune system able to control common infections aside from βHPV [23]. Patients with certain primary immunodeficiencies, and those immunocompromised by AIDS, or by the drug regimens used in solid organ transplant recipients can exhibit ‘atypical EV’, implying that a deficit in cellular immunity underlies the disease [24, 25]. Interestingly, the clinical manifestations of atypical EV can resolve upon restoration of immunity, but otherwise follow a similar course to typical EV [26, 27]. Further study of the impact of *TMC6*, *TMC8* or *CIB1* deficiency on the immune system and viral infection is needed to clarify the role of βHPV in the development of CSCC.

HPVs do not complete their life cycle in animals and this strict tropism necessitates the use of animal papillomavirus models for non-clinical studies [28]. The discovery in an immunocompromised mouse of skin warts loaded with a murine papillomavirus (MusPV, now renamed MmuPV1) has been important to the study of immune control of papillomaviruses because of the availability of many genetic knockout mice [29, 30]. MmuPV1 induces persistent papillomas in immunodeficient nude or SCID mice, but not in common immunocompetent laboratory strains unless CD3+ T cells are depleted [31, 32]. Interestingly, like the βHPV, MmuPV1 lacks E5, and their E6/E7 activate common pathways. A portion of the nude mice infected with MmuPV1 developed CSCC [33, 34], as did mice on longterm immunosuppression with cyclosporine [35]. This is consistent with atypical EV. UV light was not necessary for skin carcinogenesis in either MmuPV1-infected model and viral transcripts were present in the SCC, likely associated with viral integration [36]. However, with repeat passage in nude mice, MmuPV1 expression can be lost, suggesting that the malignant phenotype is virus independent [35].

MmuPV1 fails to induce disease in most inbred mouse strains and is rapidly cleared [31, 32, 37]. However, MmuPV1 can persist after cutaneous and intra vaginal challenge of the FVB strain of mice [38–40]. Exposure to UV radiation enhanced viral persistence and the development of CSCC, reflecting a systemic immunosuppression and enhanced mutagenesis [38, 39]. Longterm treatment with estrogen promoted the development of cervicovaginal SCC in MmuPV1-challenged FVB mice, and this was enhanced by UV exposure and its associated immunosuppressive effects [40]. Here we show that genetic knockouts *Tmc6* or *Tmc8* enhance the persistence of MmuPV1 infection in FVB mice, and explore their potential as a model of typical EV in which to examine their impact on viral replication, immune recognition and carcinogenesis.

## Results

The selective susceptibility of EV patients to βHPV disease has been proposed to reflect a disruption of intrinsic immunity mediated by the CIB1-TMC6-TMC8 protein complex in keratinocytes as a result of biallelic null mutations of *CIB1*, *TMC6*, or *TMC8* that permit elevated levels of viral replication [17, 41]. To explore whether occurs in the mouse system, we infected keratinocytes cultured from the tails of naive *Tmc6*^-/-^, *Tmc8*^-/-^ or wildtype FVB mice with MmuPV1 and measured early viral transcript levels 72 h later. The *Tmc8*^-/-^ and wildtype keratinocytes supported similar levels, but the MmuPv1 transcript level was lowest in the *Tmc6*^-/-^ keratinocytes (**S1A–S1C Table**). This suggests that Tmc6 and Tmc8 are not restriction factors for MmuPV1 infection of murine keratinocytes, at least *in vitro*, as their loss would otherwise result in higher levels of MmuPV1 transcripts.

### Similar levels of viral transcription early after vaginal challenge of *Tmc6*^-/-^, *Tmc8*^-/-^ and wildtype FVB mice with MmuPV1, but reduced viral control by knockout animals thereafter

**Fig 1:**
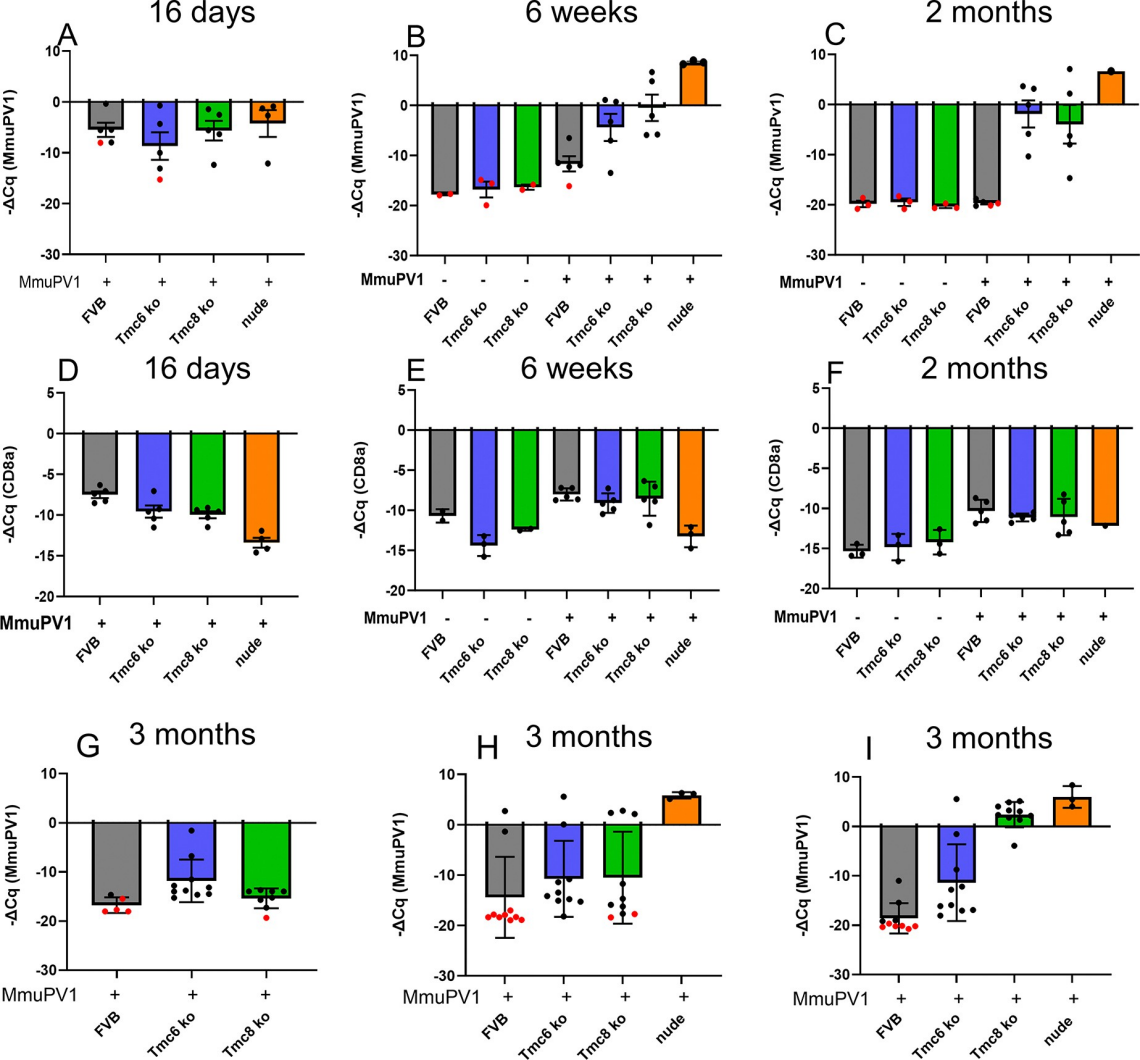
RT-PCR analysis of MmuPV1 and *Cd8a* transcripts in vaginal samples of wildtype, *Tmc6*^-/-^, *Tmc8*^-/-^ and nude mice after MmuPV1 challenge. Female *Tmc6*^-/-^, *Tmc8*^-/-^ and wildtype FVB mice (n = 5/group, aged 2–3.5 months) or age-matched nude mice (as a positive control for each challenge site) were challenged intravaginally with 20μL crude MmuPV1 wart extract. An additional 3 mice per group of male and female *Tmc6*^-/-^, *Tmc8*^-/-^ mice and wildtype mice that did not receive any challenge were included as a naïve negative control. To confirm that the vaginal challenge was successful, a vaginal brushing was harvested at day 16 post challenge (**A,D**), and a second at week 6 (**B,E**). RNA was purified from the vaginal brushings and levels of MmuPV1 (**A-C**), and *Cd8a* (**D-F**) transcripts assessed by qRT-PCR. At month 2, all animals were euthanized, and the vaginal tissue was removed and split in two. RNA was extracted from half for analysis for levels of MmuPV1 (**C**), and *Cd8a* (**F**) transcripts by qRT-PCR, and the remainder was fixed and processed for in situ hybridization (**S1 and S2 Figs**). Samples where no signal was detected at 40 cycles of qRT-PCR were assigned a value of 40 (indicated with a red dot). All qPCR data was presented as -ΔCq (-[MmuPV1 Cq–Capzb Cq]) or -ΔCq (-[CD8a Cq–Capzb Cq]) with standard error. Note, a higher, more positive number corresponds to more transcript detected. (**G**) *Tmc6*-/- (n = 10), *Tmc8*-/- (n = 10) and wildtype (n = 5) FVB mice (males, aged 1–2.5 months) were challenged on the ear with 2.8x10^10^ vge MmuPV1. At 3 months post-challenge the mice were sacrificed and the presence of MmuPV1 transcript in RNA extracted from the challenged ear was determined by qRT-PCR. MmuPV1 transcript was detected in 10/10 *Tmc6*-/- mice, 9/10 *Tmc8*-/- mice and 1/5 wildtype FVB mice. (**H, I**) *Tmc6*-/- (n = 10), *Tmc8*-/- (n = 10) and wildtype (n = 10) FVB mice (females, aged 1–2.5 months), as well as nude mice (n = 3) as a positive control, were challenged on the ear with 9.3x10^9^ vge MmuPV1 and in the vagina with 1x10^8^ vge MmuPV1. The mice were sacrificed at 3 months post-challenge and the presence of MmuPV1 transcript in RNA extracted from the challenged ear (**H**) and vagina (**I**) was determined by qRT-PCR.

To examine this question in vivo, *Tmc6*^-/-^, *Tmc8*^-/-^ mice and wildtype FVB mice (n = 5/group, aged 2–3.5 months) or age-matched nude mice (as a positive control for each challenge site) were challenged on the tail (males) or intravaginally (females) with MmuPV1. An additional 3 mice per group of male and female *Tmc6*^-/-^, *Tmc8*^-/-^ mice and wildtype mice that did not receive any challenge were included as a naïve negative control. To confirm that the vaginal challenge was successful, a vaginal brushing was harvested at day 16 post challenge, and a second at week 6. RNA was purified from the vaginal brushings and levels of MmuPV1, *Cd8a* and *Capzb* transcripts assessed by RT-PCR (**Fig 1**). Data are presented as -ΔCq, such that a higher, more positive number corresponds to more transcript detected. At day 16 post challenge, the levels of MmuPV1 transcript normalized to *Capzb* were similar in *Tmc6*^-/-^ and *Tmc8*^-/-^ mice and wildtype FVB mice (p = 0.55), suggesting that the challenges were successful and consistent. At this time point, there is little evidence of immune control based on prior work in C57BL/6 mice, and because the MmuPV1 transcript level was similar in the nude mouse positive control (**Fig 1A**). This suggests that MmuPV1 initially infects and replicates at similar levels in the genital tract of *Tmc6*^-/-^, *Tmc8*^-/-^ mice and wildtype FVB mice, arguing against a role for Tmc6 and Tmc8 as intracellular restriction factors [17].

Interestingly, the levels of *Cd8a* transcripts were subtly lower in the day 16 brushings from *Tmc6*^-/-^ (p = 0.04) and *Tmc8*^-/-^ mice (p = 0.003) as compared to wildtype FVB mice. The levels of *Cd8a* transcripts were much lower in the day 16 brushings from nude mice (which are known to have substantial reductions in CD8 T cell numbers) as compared to wildtype (p = 7x10^-5^), *Tmc6*^-/-^ (p = 0.006) and *Tmc8*^-/-^ mice (p = 0.002) animals [42].

At 6 weeks post challenge the levels of MmuPV1 transcript in the vaginal brushing from the immunodeficient nude mice were much higher (**Fig 1B**), indicative of an unrestrained expansion of the infection, as compared to wildtype mice suggesting the onset their immune response (p = 6x10^-5^). The levels of MmuPV1 transcript in the vaginal brushing from *Tmc6*^-/-^ (p = 0.05) and *Tmc8*^-/-^ (p = 0.01) mice were elevated compared to wildtype FVB at week 6, but remained well below that of the nude mice. Notably the levels of MmuPV1 transcript in the vaginal brushing from *Tmc6*^-/-^ (p = 0.17) and *Tmc8*^-/-^ (p = 0.22) mice were not significantly changed from the day 16 levels. By contrast, the level of MmuPV1 transcript in the wildtype FVB mice was lower at 6 weeks as compared to 16 days post challenge (p = 0.03). This suggests a restraint of MmuPV1 replication by *Tmc6*^-/-^ (p = 0.01) and *Tmc8*^-/-^ (p = 0.04) mice that is weaker than seen in wildtype mice at week 6. Notably, the levels of *Cd8a* transcripts in the 6 week brushings from *Tmc6*^-/-^ and *Tmc8*^-/-^ mice as compared to wildtype mice were very similar (n.s.) in mice that received MmuPV1 challenge, but were lower in the unchallenged *Tmc6*^-/-^ (p = 0.04) and *Tmc8*^-/-^ (p = 0.1) mice as compared to wildtype FVB mice. The levels of *Cd8a* transcripts were lower in non-challenged versus challenged wildtype (p = 0.01), *Tmc6*^-/-^ (p = 0.001) and *Tmc8*^-/-^ (p = 0.06) mice.

At month 2, all animals were euthanized, and the vaginal tissue was removed and split in two. RNA was extracted from half, and the remainder was fixed and processed for in situ hybridization. As anticipated, the level of MmuPV1 transcript was very high in the vaginal tissue of nude mice, although slightly lower than in the brushing at week 6 (**Fig 1**). This likely reflects the additional stromal tissue in the vaginal wall that is not infectable, whereas the brushing primarily samples the epithelium that harbors the virus. Notably, 4/5 wildtype mice showed no detectable MmuPV1 transcript (by 40 cycles) and minimal signal in one, suggesting regression of earlier infections by a delayed immune response. In contrast, robust infection was still detected in the vaginal tissues of the *Tmc6*^-/-^ (p = 0.0002) and *Tmc8*^-/-^ (p = 0.004) mice as compared to wildtype at month 2. Indeed, the levels of MmuPV1 transcripts in the samples of the *Tmc6*^-/-^ and *Tmc8*^-/-^ mice did not differ significantly across time (both n.s.). No trend of lower levels of *Cd8a* transcript was observed in the vaginal tissue of *Tmc6*^-/-^ and *Tmc8*^-/-^ mice as compared to the wildtype mice at 2 months post challenge (both n.s.), perhaps reflecting measurement of the whole tissue rather than the infected epithelium. The levels of *Cd8a* transcripts in the vaginal tissue was consistently higher in the challenged mice as compared to the naïve animals (*Tmc6*^-/-^ p = 0.005; *Tmc8*^-/-^ p = 0.003; wildtype p = 2x10^-5^). This may reflect differences in the estrous cycle as only the challenged mice were treated with Depo-provera to synchronize in diestrus [43, 44]. Alternatively, the presence of MmuPV1 infection may have attracted the CD8 T cells to the genital tract, and then even after viral clearance memory cells retained are therein. However, this trend was also evident in the vaginal brushings at week 6 (*Tmc6*^-/-^ p = 0.001; *Tmc8*^-/-^ p = 0.06; wildtype p = 0.01), suggesting they are drawn into the epithelium upon injury by brushing during the vaginal challenge process.

### Reduced viral control by *Tmc6*^-/-^ or *Tmc8*^-/-^ mice as compared to wildtype FVB mice after tail challenge with MmuPV1

For the male mice that were challenged on their tail and euthanized 2 months later, the tail skin at the challenge site was dissected and split in two. Again, RNA was extracted from half, and the remainder was fixed and processed for in situ hybridization. Only the positive control nude mouse had visible warts on the tail, and very high levels of MmuPV1 transcript were detected. Conversely, only 3/5 of the *Tmc6*^-/-^ mice and 1/5 *Tmc8*^-/-^ mice had detectable, but very low levels of MmuPV1 transcript, and none detected in the 5 wildtype mice (**S2 Table**). In contrast to challenges on the ear [45], no lesions were visible on the tails. These low levels imply that our challenge method may not be as effective in cutaneous skin of the tail and/or the virus may replicate more readily in the vaginal epithelium. While the findings are suggestive of enhanced persistence of MmuPV1 in the tail skin of *Tmc6*^-/-^ mice and *Tmc8*^-/-^ mice as compared with wildtype animals, further studies are needed to confirm this hypothesis.

The other half of the tail tissue was fixed, sectioned and stained with H&E, chromogenic immunohistochemical staining with rabbit antisera to either MmuPV1 E7 or MmuPV1 L1/L2 VLP, or chromogenic in situ hybridization with an MmuPV1 *E6/E7*-specific probe using the RNAscope system (**S1 Fig**). The tail wart on the nude mouse showed the typical morphology of a papilloma, robust nuclear staining for E7 and L1/L2 in the epithelium and high levels epithelial levels of MmuPV1 *E6/E7* transcript that was absent from normal skin. This implies that in the absence of cellular immunity, MmuPV1 replicates robustly in tail skin. No evidence of staining for MmuPV1 E7, L1/L2 or *E6/E7* transcript was evident in the sections of the tails of the *Tmc6*^-/-^, *Tmc8*^-/-^ or wildtype FVB mice that were challenged 2 months prior. In the sections of vaginal epithelium of the nude mice challenged 2 months earlier with the same dose of MmuPV1, its *E6/E7* transcript was present at significant levels, but the staining was less intense than seen in the cutaneous wart. Likewise, the immunohistochemical staining of MmuPV1 E7 and L1/L2 was also less robust than in the cutaneous lesions. This argues against poor replication in cutaneous skin as compared to the vaginal epithelium.

Weaker and less frequent staining for MmuPV1 *E6/E7* transcript was evident in the sections of the vaginal epithelium of the *Tmc6*^-/-^, *Tmc8*^-/-^ mice that were challenged 2 months prior. Interestingly, MmuPV1 *E6/E7* transcript was not visualized in the vaginal epithelium of the wildtype FVB mice at 2 months post challenge. These observations are consistent with the RT-PCR analysis of MmuPV1 transcript levels being present in *Tmc6*^-/-^, *Tmc8*^-/-^ mice, but not wildtype FVB mice, and at much lower levels than seen in nude mice at month 2 post-challenge (**S2 Fig**). Immunohistochemical staining with the antisera to either MmuPV1 E7 or L1/L2 was not convincingly above background in the sections of the vaginal epithelium of the *Tmc6*-/-, *Tmc8*-/- mice, suggesting very low expression levels resulting from partial immune control and suppression of viral transcription.

### Reduced viral control by *Tmc6*^-/-^ or *Tmc8*^-/-^ mice as compared to wildtype FVB mice after ear challenge with MmuPV1

A second study was performed in which *Tmc6*-/- (n = 10), *Tmc8*-/- (n = 10) and wildtype (n = 5) FVB mice (males, aged 1–2.5 months) were challenged on the ear with 2.8x10^10^ vge MmuPV1. At 3 months post-challenge the mice were sacrificed and the presence of MmuPV1 transcript in RNA extracted from the challenged ear was determined by qRT-PCR (**Fig 1G**). MmuPV1 transcript was detected in 10/10 *Tmc6*-/- mice, 9/10 *Tmc8*-/- mice and 1/5 wildtype FVB mice, providing further support that the virus is more persistent in the knockout animals regardless of challenge site.

A third study was performed in which *Tmc6*-/- (n = 10), *Tmc8*-/- (n = 10) and wildtype (n = 10) FVB mice (females, aged 1–2.5 months), as well as nude mice (n = 3) as a positive control, were challenged on the ear with 9.3x10^9^ vge MmuPV1 and in the vagina with 1x10^8^ vge MmuPV1 (**Fig 1H and 1I**). Ear warts appeared around 6 weeks after challenge in 2/10 mice in wildtype FVB and *Tmc6*-/- mice, 3/10 *Tmc8*-/- mice and 3/3 nude mice. The mice were sacrificed at 3 months post-challenge and the presence of MmuPV1 transcript in RNA extracted from the challenged ear was determined by qRT-PCR. MmuPV1 transcript was detected in the ear of 10/10 *Tmc6*-/- mice, 8/10 *Tmc8*-/- and 2/10 wildtype (**Fig 1H**) and in the vagina of 10/10 *Tmc6*-/- mice, 10/10 *Tmc8*-/- and 4/10 wildtype (**Fig 1I**), providing further support that the virus is more persistent in the skin of the knockout animals regardless of challenge site (tail or ear) or gender.

### Flow cytometric analyses of immunophenotype of naïve and MmuPV1-challenged wildtype, *Tmc6*^-/-^ and *Tmc8*^-/-^ mice

While CIB1 and some TMC6 is expressed in human and murine keratinocytes, TMC8 expression is barely detectable [21, 46]; this is not consistent with the hypothesis that the CIB1-TMC6-TMC8 heterotrimeric protein complex is mediating keratinocyte intrinsic immunity. By contrast, all three proteins are expressed in thymocytes of mice and the human acute T cell lymphoma line Jurkat. An alternate hypothesis is that the CIB1-TMC6-TMC8 heterotrimeric protein complex is important in mediating immune control of βHPV infection, potentially in T cell function and/or trafficking to the skin.

**Fig 2:**
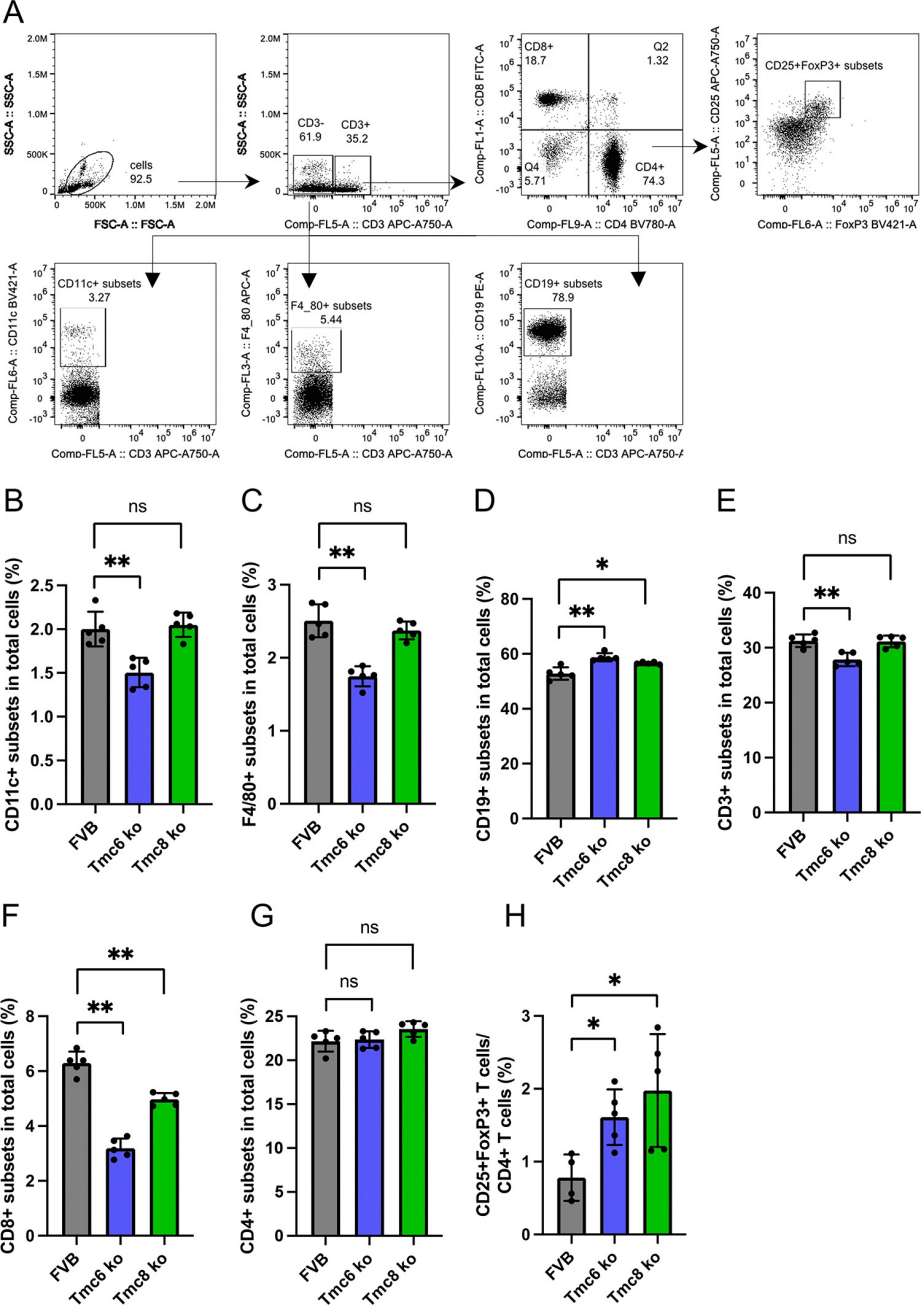
Leukocyte composition in the spleen of wildtype, *Tmc6*^-/-^, and *Tmc8*^-/-^ mice. Splenocytes were isolated from naïve wildtype (n  =  5), *Tmc6*^-/-^ (Tmc6 ko, n  =  5), and *Tmc8*^-/-^ mice (Tmc8 ko, n  =  5) of the FVB strain (7–8 week old males). Individual immune subsets were stained for flow cytometry analysis. (A) Representative flow cytometry plots illustrating the gating strategy used to identify the immune subsets in the spleen. The following parameters were examined: frequencies of (B) CD11c+ dendritic cells, (C) F4/80+ macrophages, (D) CD19+ B cells, (E) CD3+ T cells, (F) CD8+ T cells, and (G) CD4+ T cells in CD45+ leukocytes, and (H) frequencies of CD25+FoxP3+ regulatory T cells within the CD4+ T cell population.

**Fig 3:**
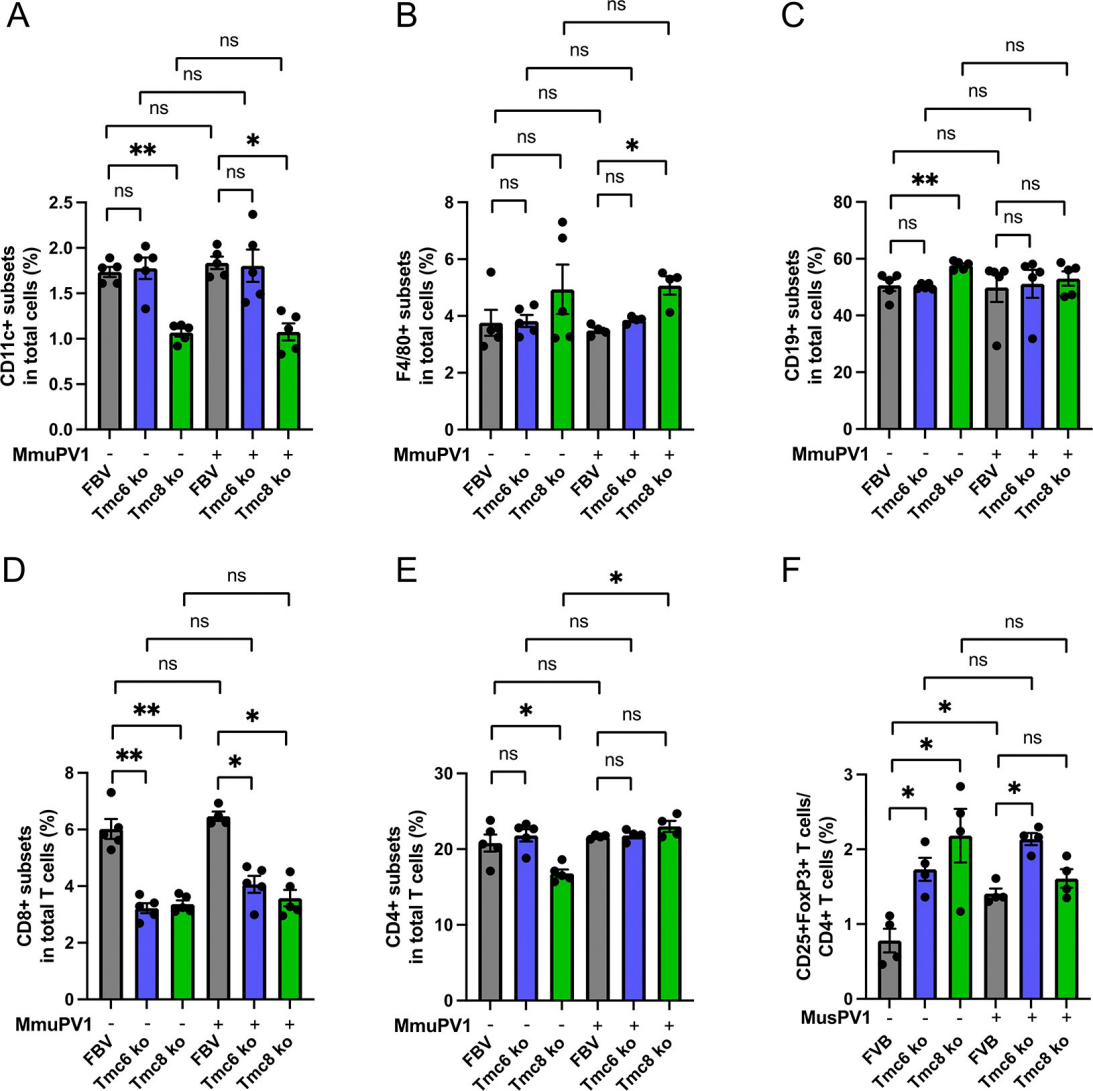
Leukocyte composition in the spleen of wildtype, *Tmc6*^-/-^, and *Tmc8*^-/-^ mice at 3 weeks after MmuPV1 challenge. Naïve wildtype, *Tmc6-/-*, and *Tmc8-/-* mice (7–10 week old female) were challenged intravaginally with 10^8^ vge MmuPV1, and sacrificed at 3 weeks post infection (n  =  5/group). The levels of viral transcript were measured in vaginal tissues and confirmed successful infection (**S1A Fig**). Splenocytes were isolated and individual immune cell populations were stained for flow cytometry analysis. The following parameters were examined: frequencies of (A) CD11c+ dendritic cells, (B) F4/80+ macrophages, (C) CD19+ B cells, (D) CD8+ T cells, and (E) CD4+ T cells in CD45+ leukocytes. (F) frequencies of CD25+FoxP3+ regulatory T cells within the CD4+ T cell population.

Several immunophenotyping studies of typical EV patients describe numbers of T cell, B cells and NK cells within normal range of the general population and with appropriate compartmentalization [17]. Although prior studies of EV patients suggested that their immune system generally remains intact, we considered whether naive *Tmc6*^-/-^ and *Tmc8*^-/-^ mice have an immune deficit as compared to wildtype animals (n = 5/group, 7–8 week old males), each in the FVB background. An initial analysis of splenocytes was performed by multiparameter flow cytometry for ratios of key immune cell populations, (**Fig 2A**) including: CD11c+ dendritic cells (**Fig 2B**), F4/80+ macrophages (**Fig 2C**), CD19+ B cells (**Fig 2D**), CD3+ T cells (**Fig 2E**), CD8+ T cells (**Fig 2F**), CD4+ T cells (**Fig 2G**), or CD4+CD25+FoxP3+ T cells as a fraction of CD4+ T cells (**Fig 2H**). The CD11c+ dendritic cell (**Fig 2B**), F4/80+ macrophages (**Fig 2C**) and CD3+ T cell fractions (**Fig 2E**) were slightly lower compared to wildtype FVB mice for the naive *Tmc8*^-/-^ but not *Tmc6*^-/-^ mice, but these differences were not consistent (**Fig 3A and 3B**). Likewise, the B cell (**Figs 2D and 3C**) and CD4+ T cell (**Fig 2G and 2E**) fractions did not consistently differ between wildtype FVB and the knockout mice. However, a consistently lower CD8+ T cell fraction (~half) was evident (**Figs 2F and 3D**) in naïve *Tmc8*^-/-^ and *Tmc6*^-/-^ mice as compared to wildtype FVB mice. Conversely, a consistently ~2-fold higher CD25+FOXP3+ fraction within CD4+ T cells was identified (**Figs 2H and 3F**) in naïve *Tmc8*^-/-^ and *Tmc6*^-/-^ mice as compared to wildtype FVB mice.

**Fig 4:**
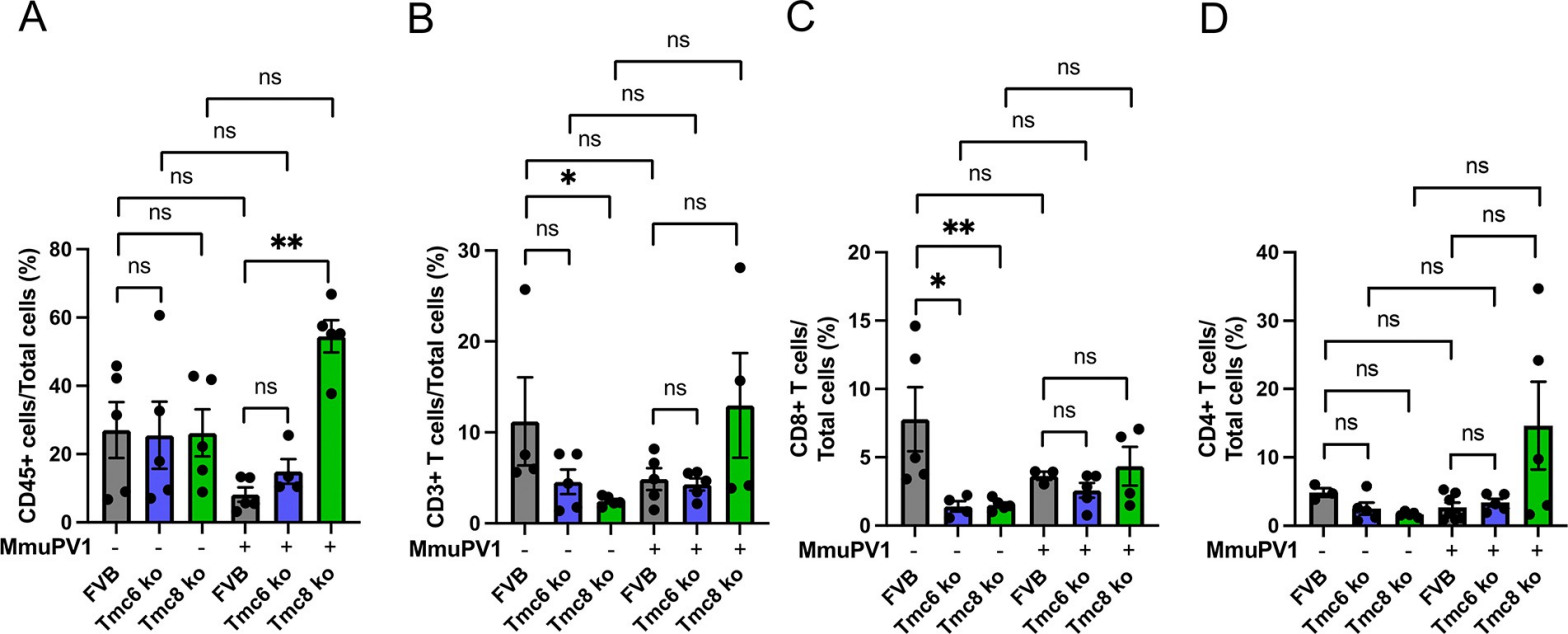
The immune landscape of vaginal brushings from wildtype, *Tmc6*^*-/-*^, and *Tmc8*^-/-^ mice, either with or without MmuPV1 challenge. Vaginal brushings were collected 17 days after MmuPV1 challenge from all groups of mice from the experiment described in **Fig 3** (n  =  5/group). Vaginal cell suspensions were stained with selected immune subsets for flow cytometry analysis. The following parameters were examined: frequencies of (A) CD45+ leukocytes, (B) CD3+ T cells, (C) CD8+ T cells, and (D) CD4+ T cells in total cells.

We sought to examine whether these differences in immune phenotype were influenced by the presence of MmuPV1 infection. Therefore, wildtype, *Tmc6*^-/-^ and *Tmc8*^-/-^ mice (n = 5/group, 7–10 week old females) were challenged intra-vaginally with 10^8^ vge of MmuPV1 and compared to mock-challenged mice (**Fig 3**). A vaginal sample was obtained at 17 days post infection (see **Fig 4**), and the mice euthanized at week 3. The success of the challenge was confirmed by measuring MmuPV1 early transcript in vaginal tissue collected at 3 weeks (**S3A Fig**). Interestingly, higher levels of MmuPV1 transcript were evident in the knockout animals (*Tmc6*^*-/-*^ p = 0.06; *Tmc8*^-/-^ p = 0.0004) at week 3. Differences compared to mock-challenged mice in the levels of dendritic cells (**Fig 2B versus 3A**) and macrophages (**Fig 2C versus 3B**) in spleens were not consistent between experiments, and were not impacted at 3 weeks after MmuPV1 challenge. B cell populations did not significantly differ between wildtype and either *Tmc6*^-/-^ or *Tmc8*^-/-^ mice, or 3 weeks after MmuPV1 challenge (**Fig 3C**), so differences observed in naïve animals were not maintained. CD8+ T cells were a significantly lower fraction in total T cells in naïve mice (**Fig 2F**) and this remained consistent at 3 weeks after MmuPV1 challenge (**Fig 3E**). Likewise, mean levels of *Cd8a* transcripts were also lower in the vaginal tissues of knockout mice at 3 weeks both in naïve mice (p = 0.03 for *Tmc6*^-/-^, p = 0.02 for *Tmc8*^-/-^ mice) as well as 3 weeks after challenge (p = 0.03 for *Tmc6*^-/-^, p = 0.001 for *Tmc8*^-/-^ mice; **S3B Fig**). CD4+ T cells as a percent of total cells was not different between wildtype and knockout mice at 3 weeks after MmuPV1 challenge (**Fig 3F**) or in naïve mice (**Fig 2G**), although some difference was seen in the mock-challenged animals (**Fig 3E**). Interestingly, CD4+CD25+FoxP3+ T cells as a fraction of CD4+ T cells (**Fig 3H**) were higher in the splenocytes of *Tmc6*^-/-^ and *Tmc8*^-/-^ mice than wildtype animals both for mock-challenged (**Fig 3F**) and naïve mice (**Fig 2H**), but this difference was less clear at 3 weeks post-challenge (**Fig 3F**).

Since the MmuPV1 infection is in the vaginal epithelium, we examined its impact on immune cell subsets locally using the vaginal brushings collected from the mice of **Fig 3** at 17 days after MmuPV1 challenge. Cells released from the brushes were stained for selected immune cell subsets and analyzed by flow cytometry as a fraction of total cells (**Fig 4**). The number of CD45+ leukocytes (**Fig 4A**) and CD3+ T cells (**Fig 4B**) present in the vaginal brushing was generally similar in the mock-challenged wildtype, *Tmc6*^-/-^ and *Tmc8*^-/-^ mice, and did not significantly differ in the mice 17 days post challenge. A caveat is that the immunophenotype analysis in the vaginal track brushings was more variable than for splenocytes because of the relatively fewer cells available for staining. Interestingly, the CD8+ T cell population was higher in the vaginal brushing in mock-challenged wildtype mice than from the *Tmc6*^-/-^ and *Tmc8*^-/-^ mice (**Fig 4C**), consistent with systemic findings (**Figs 2F and 3E**) and measurement of *Cd8a* mRNA in vaginal tissues. However, this difference was lost in the mice at 3 weeks post challenge (**Fig 4C**). No such difference was seen in the CD4+ T cell subset (**Fig 4D**).

**Fig 5:**
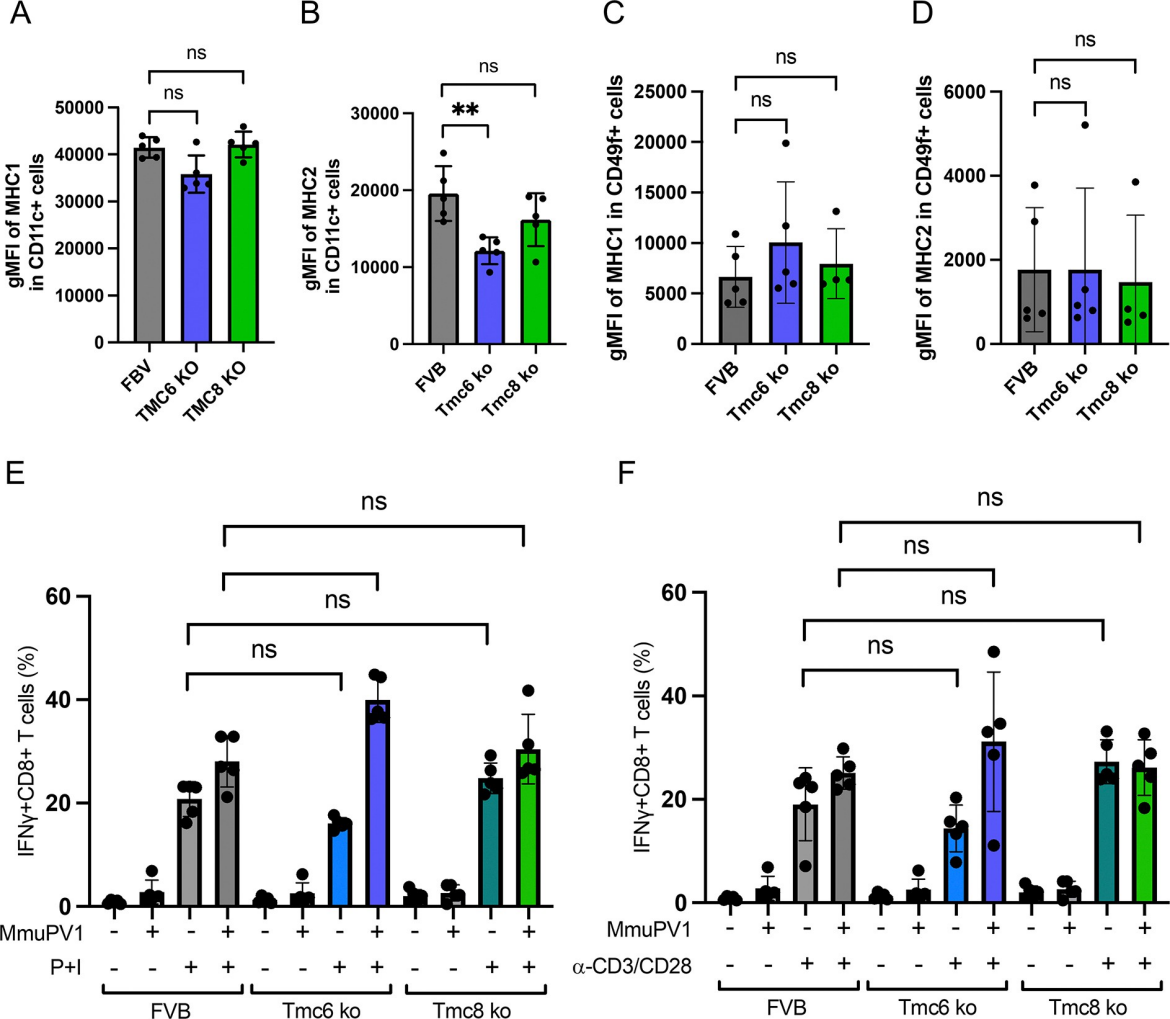
MHC complex expression on CD11c+ dendritic cells and interferon-γ production by CD8+ T cells of naive wildtype, *Tmc6*^-/-^, and *Tmc8*^-/-^ mice. Flow cytometric analysis of the surface expression of MHC class I and II molecules and CD11c on splenocytes of naïve wildtype, *Tmc6*^-/-^, and *Tmc8*^-/-^ mice (n  =  5/group, 7–8 week old males). (A) The gMFI values for MHC class I on CD11c+ cells. (B) The gMFI values for MHC class II on CD11c+ cells. (C-D) Wildtype, *Tmc6*^-/-^, and *Tmc8*^-/-^ mice (10–14 week old females) were challenged with 3.7x10^10^ vge MmuPV1(n = 5/group) or naïve (n  =  2 wildtype and 3 *Tmc6* -/-). Keratinocytes were collected 2 weeks after immunization through samplings using vaginal swabs and stained with antibodies against CD49f, MHC class I, and MHC class II. FACS analysis of the expression of MHC class I and II molecules on CD49f+ keratinocytes of FVB, *Tmc6*^-/-^, and *Tmc8*^-/-^ mice. (C) The gMFI values for MHC class I on CD49f+ cells. (D) The gMFI values for MHC class II on CD49f+ cells. (E-F) Splenocytes were isolated from wildtype, *Tmc6*^-/-^, and *Tmc8*^-/-^ mice (n  =  5/group, 7–10 week old female) at 3 weeks post challenge intravaginally with 10^8^ vge MmuPV1 or mock challenge (see **Fig 2**). The splenocytes were stimulated with Phorbol 12-myristate 13-acetate and Ionomycin (P+I) in (E) or anti-CD3 plus anti-CD28 antibody (F) for 24 h. Stimulated cells were analyzed for IFNγ expression in CD8+ T cells by surface and intracellular staining and flow cytometry. Statistical analysis utilized the Student’s T-test. Representative gating is shown in **S4 and S5 Figs**.

Within cells, Tmc6 and Tmc8 reside in a similar vesicular compartment (endoplasmic reticulum) as the site MHC-I and MHC-II loading, and therefore may impact antigen presentation. Indeed, E5 both interacts with CIB1, TMC6 and TMC8 [17], and also down-regulates MHC-I and MHC-II surface display, further suggesting such a link [47]. As a surrogate measure of antigen presentation, we assessed the surface levels of MHC-I (**Fig 5A**) and MHC-II molecules (**Fig 5B**) on dendritic cells harvested from spleens of naïve wildtype, *Tmc6*^-/-^ and *Tmc8*^-/-^ mice. Likewise, we assessed the surface levels of MHC-I (**Fig 5C**) and MHC-II molecules (**Fig 5D**) on the surface of keratinocytes harvested from brushings of the vaginal tract of naïve wildtype mice and from the *Tmc6*^-/-^ and *Tmc8*^-/-^ FVB mice. The mice were each challenged with 3.7x10^10^ vge MmuPV1 or as negative control, naïve, unchallenged mice were used. MmuPV1 transcripts were detected in all challenged mice 2 weeks post challenge; naïve mice did not have any MmuPV1 transcripts (**S3C Fig**). While some differences between wildtype and either *Tmc6*^-/-^ or *Tmc8*^-/-^ mice were noted in naïve animals (**Fig 5A and 5B**), this was not consistent in keratinocytes harvested from either mock or MmuPV1-challenged mice (**Fig 5C and 5D**). This might reflect experimental variation/low cell numbers, or possibly that inflammation caused by the vaginal challenge itself eliminates the differences observed in the naïve animals (**Fig 5A–5D**).

### Activation, proliferation and zinc levels in CD8 T cells derived from naïve and MmuPV1-challenged *Tmc6*^-/-^, *Tmc8*^-/-^ and wildtype FVB mice

Normal proliferative capacity in response to anti-CD3 stimulation was observed in T cells of typical EV patients [48] [17]. Initially, we examined the status of T cell effector function in splenocytes from wildtype, *Tmc6*^-/-^ or *Tmc8*^-/-^ mice harvested 3 weeks after mock-challenge or vaginal challenge with 10^8^ vge MmuPV1 (n = 5/group, from **Fig 3**). The splenocytes were stimulated with PMA and ionomycin (**Fig 5E**), or anti-CD3 and anti-CD28 antibodies (**Fig 5F**) for 24 h and expression of interferon-γ (IFNγ) detected by flow cytometry. The CD8+ T cells of wildtype, *Tmc6*^-/-^ or *Tmc8*^-/-^ mice responded similarly to activation by either stimulus, and these responses were not consistently impacted by MmuPV1 infection.

**Fig 6:**
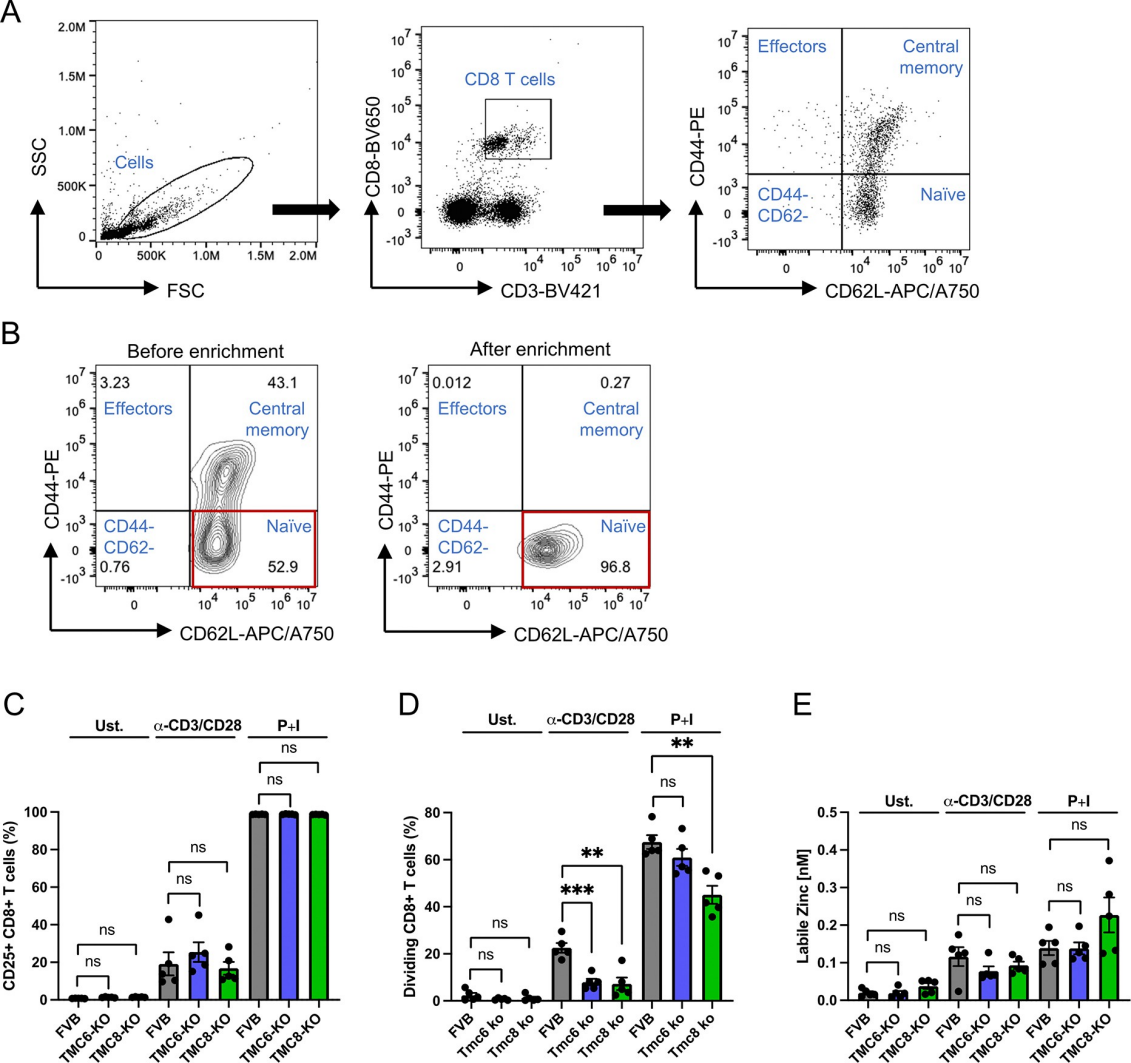
Activation, proliferation, and intracellular zinc levels of CD8 T cells of wildtype, *Tmc6*^-/-^, and *Tmc8*^-/-^ mice. Naïve CD8 T cells were magnetically isolated from the spleens of wildtype, *Tmc6*^-/-^, and *Tmc8*^-/-^ mice (n = 5/group, 9–13 week old female), and labeled with CellTrace Far Red. The CD8+ T cells were activated through either PMA/Ionomycin or anti-CD3/anti-CD28 for 24 h. Statistical analysis utilized the Student’s T-test. (A) Representative gating strategy to analyze the expression of CD3, CD8, CD44, and CD62L on T cells before enrichment. (B) The flow cytometric plot shows the expression of CD62L and CD44 before and after enrichment. (C) The percentage of CD25+CD8+ T cells in total CD8 T cells. (D) The percentage of dividing CD8+ T cells. (E) The concentration of free zinc was measured using FluoZin-3. Representative gating is shown in **S4 and S5 Figs**.

We also compared *in vitro* activation and proliferation of CD8+ T cells purified from *Tmc6*^-/-^ or *Tmc8*^-/-^ and wildtype FVB mice (n = 5/group, 9–13 week old females). Naïve CD8+ T cells were separated from the spleens of naïve mice, their purity assessed by flow cytometry (**Fig 6A and 6B**), and labeled with CellTrace Far Red dye. The labeled naïve CD8+ T cells were stimulated with PMA and ionomycin, or anti-CD3 and anti-CD28 antibodies (**6C, 6D**) for 24 h and surface expression of CD25 (**Fig 6C**) and CellTrace Far Red fluorescence (**Fig 6D**) detected by flow cytometry. As seen for IFNγ production, surface levels of the activation marker CD25 were similarly elevated in CD8+ T cells purified from *Tmc6*^-/-^ or *Tmc8*^-/-^ and wildtype FVB mice in response to anti-CD3 and anti-CD28 antibodies or PMA and ionomycin, although the response to the latter was much higher (**Fig 6C**). Interestingly, the fraction of *Tmc6*^-/-^ or *Tmc8*^-/-^ CD8 T cells that were dividing was significantly lower after stimulation with anti-CD3 and anti-CD28 antibodies as compared to wildtype. A similar trend was also evident after stimulation with PMA and ionomycin, although it did not reach significance for *Tmc6*^-/-^ CD8 T cells (**Fig 6C**).

Prior studies have suggested higher cellular levels of zinc are present in EBV-transformed lymphoblastoid cell lines derived from EV as compared to healthy individuals [46]. Thus we compared basal cellular zinc levels, and levels upon *in vitro* activation of CD8+ T cells purified from *Tmc6*^-/-^ or *Tmc8*^-/-^ and wildtype FVB mice (n = 5/group, 9–13 week old females). Further, activation of wildtype murine CD8+ T cells with anti-CD3 and anti-CD28 antibodies for 24 h elicited an increase in cellular levels of free zinc [46]. We stimulated naïve CD8+ T cells with PMA and ionomycin, or anti-CD3 and anti-CD28 antibodies for 24 h and measured cellular levels of free zinc using FluoZin-3 fluorescence (**Fig 6E**) detected by flow cytometry, as described by [46]. The cellular level of zinc was similarly low in naïve CD8 T cells purified from *Tmc6*^-/-^ or *Tmc8*^-/-^ and wildtype FVB mice. An increase was FluoZin-3 fluorescence was evident at 24 h after activation by anti-CD3 and anti-CD28 antibodies or PMA and ionomycin, but the levels were not consistently different in the knockout versus the wildtype CD8 T cells. However, a caveat is that these levels are near the lower limit of detection with FluoZin-3 dye.

### Rapid control of cutaneous vaccinia infection by wildtype, *Tmc6*^-/-^ and *Tmc8*^-/-^ mice

Antibody titers against common viral infections were observed in patients with typical EV in ranges consistent with the general population, and clinical reports suggest no abnormal susceptibility to infections other than cutaneous βHPV [17, 48]. To assess whether control of an unrelated viral infection of skin is normal (or there is a generalized failure of the immune system function) in *Tmc6*^-/-^ and *Tmc8*^-/-^ mice as compared to wildtype FVB mice, their ability to control vaccinia virus (VV) after percutaneous challenge was compared (**Figs S8**, **and 7**). Mice (5/group) were challenged on the tail by skin scarification with a recombinant vaccinia virus expressing firefly luciferase (VV-luc). The infection was imaged and quantified by injection of luciferin and imaging bioluminescence using an IVIS machine on days 1, 6, 10 and 14 (**S8 Fig**). VV-luc infection peaked at a similar level around 1 week and was controlled in all mice by 2 weeks, although the signal was lowest in the *Tmc6*^-/-^ mice. In a repeat experiment with 10 mice/group and imaging on days 2, 6, 9 and 12, the VV-luc infection again peaked at a similar level at around one week and was controlled in all animals by the second week. While no clear differences were identified between wildtype and *Tmc6*^-/-^ and *Tmc8*^-/-^ mice (**Fig 6**), the signal was again lowest and slightly delayed in the *Tmc6*^-/-^ mice. In addition to CD4, CD8 T cells and macrophages [49], an antibody response is obligatory for recovery from a primary poxvirus infection [50]. Thus, we examined levels of neutralizing antibodies post VV-luc challenge. Analysis of sera obtained from the mice 2 weeks post VV-luc challenge showed that all three groups of mice generated low titers of VV neutralizing antibody. The slightly higher titer (1:40) in *Tmc8*^-/-^ and wildtype mice than for *Tmc6*^-/-^ mice (1,20) may account for the slightly delayed clearance of VV-luc by *Tmc6*^-/-^ mice (**S9 Fig**).

**Fig 7:**
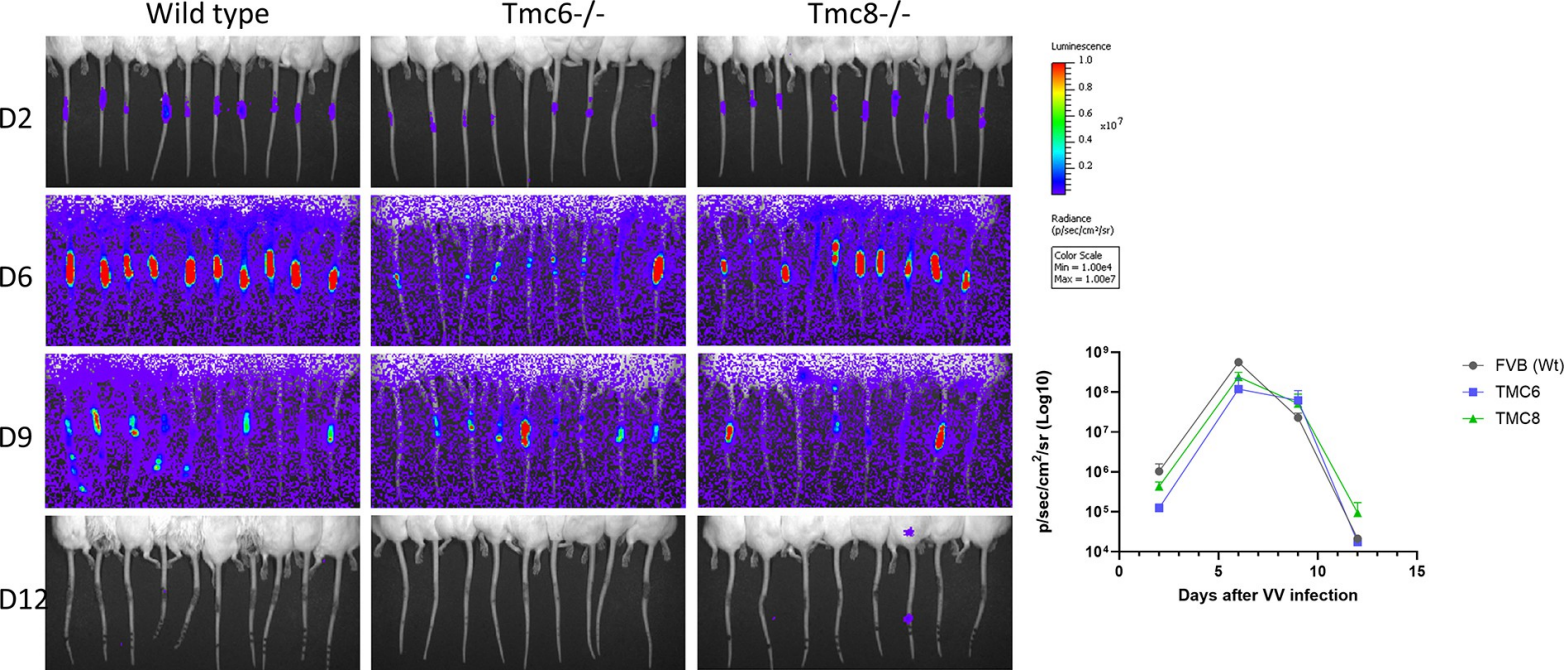
Control of vaccinia virus expressing luciferase after percutaneous challenge of *Tmc6*^-/-^, *Tmc8*^-/-^ or wildtype FVB mice. Infectivity in wildtype, *Tmc6*^-/-^ and *Tmc8*^-/-^ FVB mice of recombinant vaccinia virus expressing luciferase (VV-luc) was assessed in 6–8 week old *Tmc6*^-/-^, *Tmc8*^-/-^ and wildtype FVB male mice (n = 10/group). Briefly, mice were anesthetized and 5x10^5^ pfu (5μL) of recombinant vaccinia virus expressing luciferase (VV-luc) was applied to tail skin 1 cm from the base of the tail on day 0. The skin area was then gently scratched 15 times with a bifurcated needle. Mice were imaged by IVIS Spectrum in vivo imaging system series 2000 (PerkinElmer) at days 2, 6, 9 and 12. Total photon counts were quantified in the tumor site by using Living Image 2.50 software (PerkinElmer). Luminescence imaging from each group is presented, quantification of luminescence signal in the region of interest (ROI) shown graphically.

### DNA Vaccination to treat MmuPV1 infection

**Fig 8:**
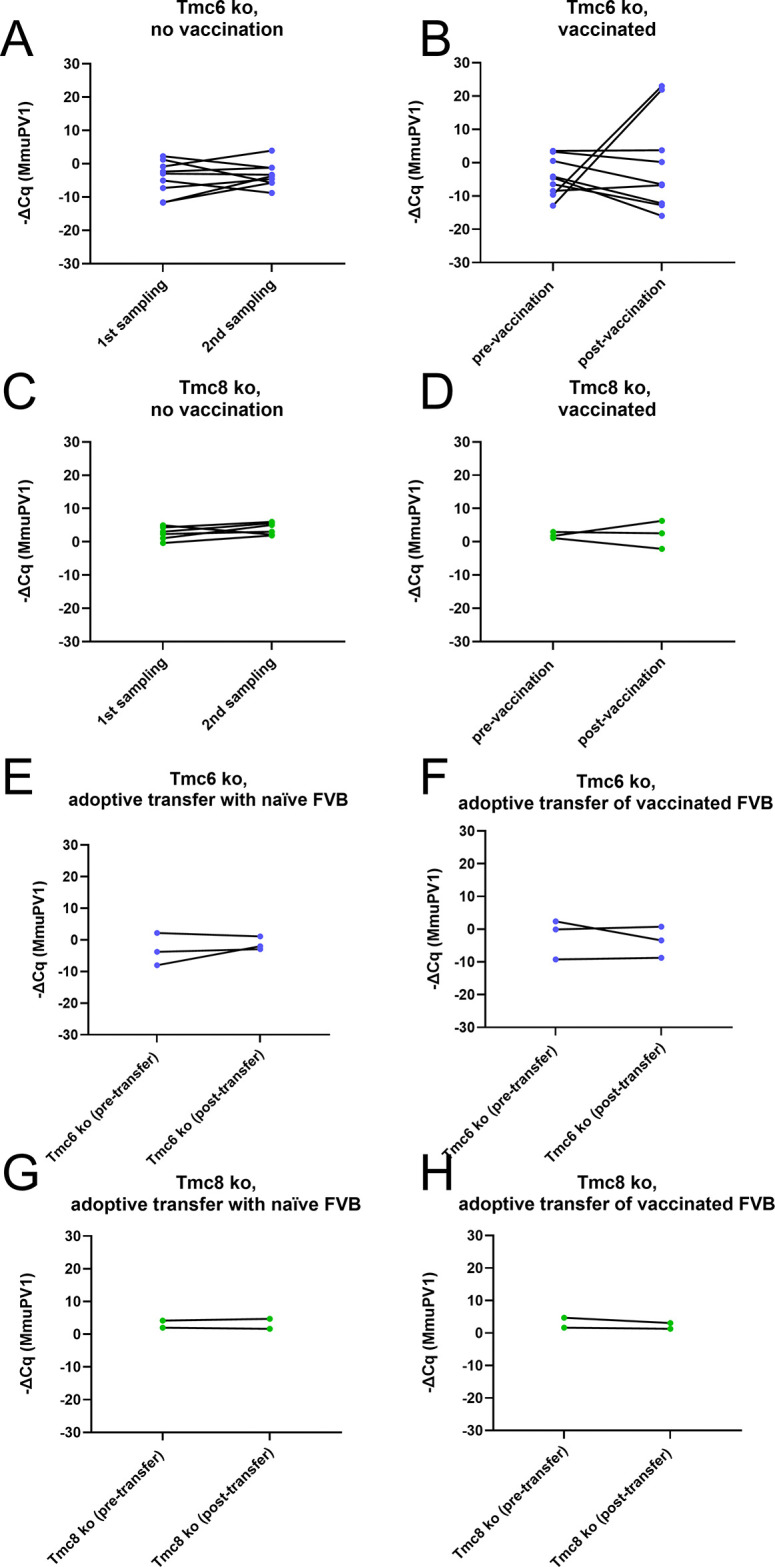
Impact of adoptive transfer of splenotypes from naïve or vaccinated wildtype FVB mice on MmuPV1 transcript levels in *Tmc6*^-/-^ or *Tmc8*^-/-^ FVB mice. *Tmc6*^-/-^ (n = 18), *Tmc8*^-/-^ (n = 9) FVB mice (females, aged 3 months) were challenged intravaginally with 5.1x10^11^ vge MmuPV1, and their vagina was first sampled at 5 weeks post-challenge (**A-D**). Two weeks later, 9/18 of the challenged *Tmc6*^-/-^ mice (**B**) and 3/9 of the challenged *Tmc8*^-/-^ mice (**D**), as well as 5 of 10 additional wildtype FVB mice (female, 3 months old), were then administered 15 μg of hCRT-mE6mE7mL2 DNA intramuscularly three times at 2 week intervals with electroporation. The remaining 7/14 *Tmc6*^-/-^ mice (**A**), 6/9 of *Tmc8*^-/-^ mice (**C**) and 5/10 wildtype FVB mice were not vaccinated. Two weeks after the vaccination was completed, serum was collected and analyzed in **Fig 9A**. A second vaginal sampling from the challenged mice was taken 4 weeks after the final vaccination (**A-D**), and the levels of MmuPV1 transcript present in the paired samples was compared by qRT-PCR. There was no significant difference by paired T test in the level of MmuPV1 transcript present before versus after vaccination in the vaccinated (n = 9, p = 0.24, **B**) or unvaccinated *Tmc6*^-/-^ mice (n = 9, p = 0.24, **A**), or in the vaccinated (n = 3, p = 0.46, **D**) or unvaccinated *Tmc8*^-/-^ mice (n = 6, p = 0.11, **C**). Splenocytes (10^6^) from 5 naïve wildtype FVB mice were transferred into 3 of the *Tmc6*^-/-^ (**E**) and 2 of the *Tmc8*^-/-^ mice (**G**) from the above experiment. Likewise, splenocytes (10^6^) from 5 wildtype FVB mice vaccinated with hCRT-mE6mE7mL2 DNA were transferred into 3 of the *Tmc6*^-/-^ (**F**) and 2 of the *Tmc8*^-/-^ mice (**H**) from the above experiment. One month after adoptive transfer, the vaginal tracts were sampled to measure MmuPV1 transcript levels of all ten mice (**E-H**). No significant change in the levels of MmuPV1 transcript from before versus a month after adoptive transfer were observed, either in the *Tmc6*^-/-^ or the *Tmc8*^-/-^ mice, or either those that received splenocytes from wildtype FVB mice previously vaccinated with hCRT-mE6mE7mL2 DNA (**F,H**) or not (**E,G**).

**Fig 9:**
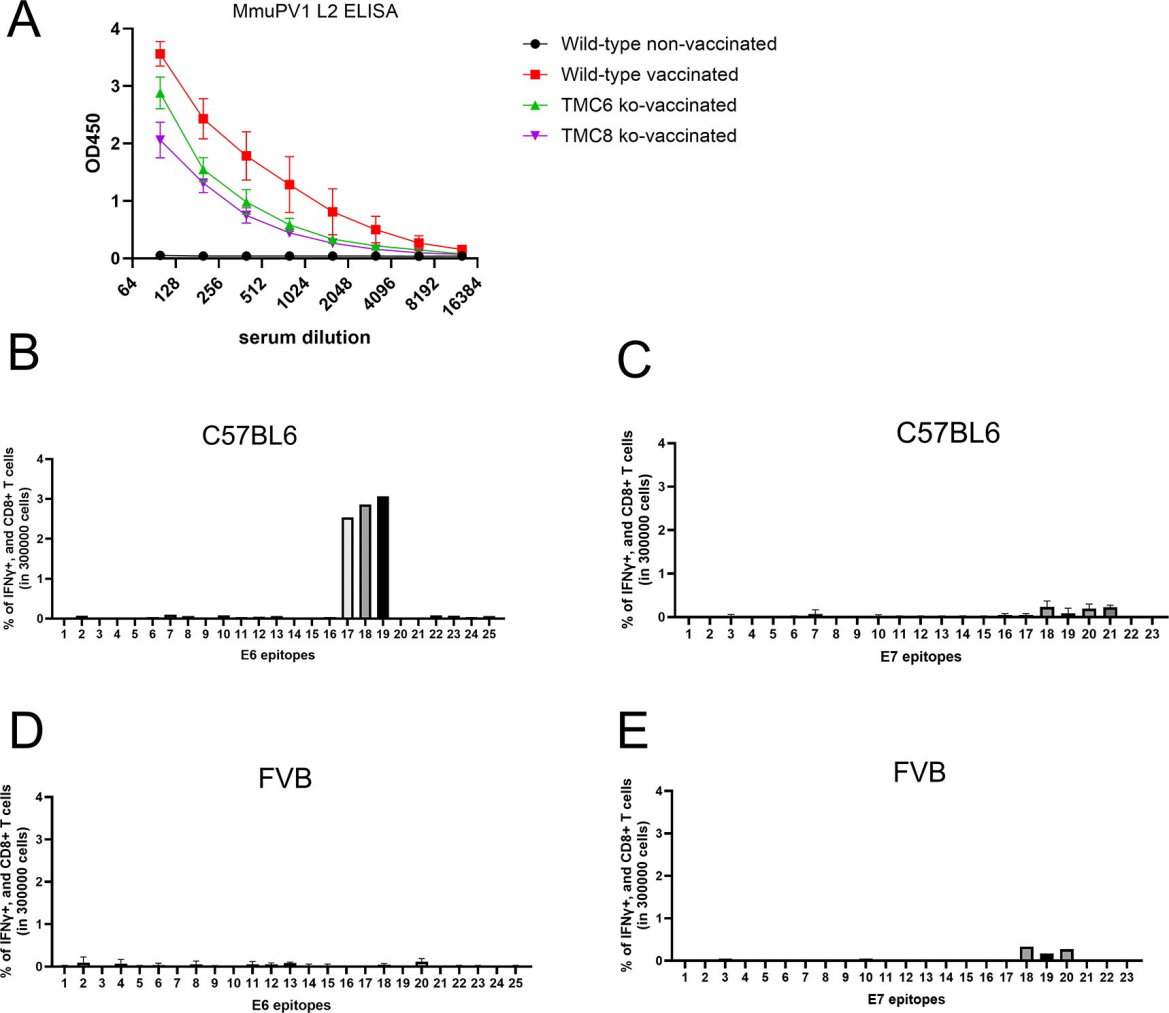
L2-specific Antibody and E6/E7-specific CD8+ T cell responses of FVB mice vaccinated with hCRT-mE6mE7mL2 DNA. *Tmc6*^-/-^, *Tmc8*^-/—^and wildtype female FVB mice were injected intramuscularly with 15μg of hCRT-mE6mE7mL2 DNA vaccine followed by electroporation on days 1, 15 and 29. One month after the final vaccination, sera were collected. Sera were collected were also collected from unvaccinated wildtype female FVB mice as a negative control. Sera (n = 3/group) were analyzed by ELISA for reactivity against MmuPV1 L2-6His (**A**). Briefly, 1μg/ml of MmuPV1 L2 protein in PBS was coated on BRANDplates microplates overnight at 4°C. After 16 h, the plates were washed, blocked with eBioscience ELISA/ELISPOT Diluent, and added serial 2-fold dilution of serum for two hours at room temperature. Goat anti-mouse IgG-HRP secondary antibody was added at 1:5000 dilution for 1 hour, followed by TMB substrate. The OD at 450nm was determined by 800 TS Absorbance Reader (BioTek Instruments, Inc). Characterization of MmuPV1 E6 (**B,D**) and E7 epitopes (**C,E**) recognized by CD8+ T cells of C57BL/6 (**C,D**) or FVB mice. Mice (n = 3) were injected intramuscularly with 15μg of hCRT-mE6mE7mL2 DNA vaccine followed by electroporation on days 1, 8 and 15. One week after the final vaccination, splenocytes were collected. To determine the epitopes, a panel of 20mer peptides, each overlapping by 15 amino acids were incubated with splenocytes in the presence of Golgi plug for 16 hours. Splenocytes stimulated with eBioscience Cell Stimulation Cocktail was used as a positive control. The cells were stained for interferon-γ and CD8, and analyzed by flow cytometry using a CytoFLEX S (Beckman) and data were analyzed by FlowJo software. Percentage of interferon-γ producing CD8+ T cells is presented.

We previously showed that administration of a naked DNA vaccine that expresses human calreticulin (hCRT) fused with MmuPV1 E6, E7, and amino acids [aa] 11 to 200 of L2 (hCRT-mE6mE7mL2) intramuscularly followed by in vivo electroporation successfully triggered the regression of warts on the tails of MmuPV1-challenged SKH-1 mice. Regression was associated with a robust CD8 T cell response to MmuPV1 E6 amino acids 90 to 99 (KNIVFVTVR) and a weaker response to E7 aa 76–95. To examine whether DNA vaccination could treat a persistent MmuPV1 infection, *Tmc6*^-/-^ (n = 18), *Tmc8*^-/-^ (n = 9) FVB mice (females, aged 3 months) were first challenged intravaginally with 5.1x10^11^ vge MmuPV1, and an additional cohort of 10 wildtype FVB mice (females) was not challenged. Their vagina was sampled at 5 weeks post-challenge. Two weeks later, 9/18 of the challenged *Tmc6*^-/-^ mice and 3/9 of the challenged *Tmc8*^-/-^ mice, as well as an additional 5/10 wildtype FVB mice (female, 3 months old), were then administered 15 μg of hCRT-mE6mE7mL2 DNA intramuscularly three times at 2 week intervals with electroporation (the remaining 7/14 *Tmc6*^-/-^ mice, 6/9 of *Tmc8*^-/-^ mice and 5/10 wildtype FVB mice were not vaccinated). Two weeks after the vaccination was completed, serum was collected. A second vaginal sampling was taken 4 weeks after the final vaccination. The levels of MmuPV1 transcript present in the paired samples was compared (**Fig 8A–8D**). There was no significant difference by paired T test in the level of MmuPV1 transcript present before versus after vaccination in the vaccinated (n = 9, p = 0.24, **Fig 8B**) or unvaccinated *Tmc6*^-/-^ mice (n = 9, p = 0.24, (**Fig 8A**), or in the vaccinated (n = 3, p = 0.46, **Fig 8D**) or unvaccinated *Tmc8*^-/-^ mice (n = 6, p = 0.11, **Fig 8C**). An ELISA was performed on the sera collected 2 weeks after vaccination to detect antibody specific to MmuPV1 L2 (**Fig 9A**). All vaccinated mice developed L2-specific serum antibodies, confirming successful vaccination and implying that such antibodies have no impact on pre-existing infection. The L2-specific antibody titers of the knockout mice were 100, and 400 for the wildtype FVB mice (**Fig 9A**).

### Adoptive transfer to treat MmuPV1 infection

Since wildtype FVB mice better control and clear MmuPV1 infections, we reasoned that adoptive transfer of splenocytes from wildtype FVB mice to MmuPV1-infected *Tmc6*^-/-^ or *Tmc8*^-/-^ mice might trigger viral clearance. Furthermore, we hypothesized that adoptive transfer of splenocytes from wildtype FVB mice that had previously received the hCRT-mE6mE7mL2 DNA vaccination regimen might more effectively induce clearance of MmuPV1 from *Tmc6*^-/-^ or *Tmc8*^-/-^ mice. Therefore, 5x10^6^ splenocytes from each of 5 naïve wildtype FVB mice were transferred into 3 of the *Tmc6*^-/-^ and 2 of the *Tmc8*^-/-^ mice from the above experiment. Likewise, 5x10^6^ splenocytes from each of 5 hCRT-mE6mE7mL2 DNA-vaccinated wildtype FVB mice were transferred into 3 of the *Tmc6*^-/-^ and 2 of the *Tmc8*^-/-^ mice from the above experiment. One month after adoptive transfer, the vaginal tracts were sampled to measure MmuPV1 transcript levels of all ten mice (**Fig 8E–8H**). No significant change in the levels of MmuPV1 transcript from before versus a month after adoptive transfer were observed, either in the *Tmc6*^-/-^ or the *Tmc8*^-/-^ mice, or either those that received splenocytes from wildtype FVB mice previously vaccinated with hCRT-mE6mE7mL2 DNA (**Fig 8F and 8H**) or not (**Fig 8E and 8G**). These vaccinated mice had all developed L2-specific antibodies which suggests that they were successfully vaccinated (**Fig 9A**).

### Mapping of CD8 T cells responses to MmuPV1 E6 and E7

To explore the reasons for the failure of the wildtype FVB mouse splenocytes obtained after vaccination to induce clearance of MmuPV1 from the *Tmc6*^-/-^ or *Tmc8*^-/-^ mice after adoptive transfer, we measured the CD8 T cell response. *Tmc6*^-/-^, *Tmc8*^-/-^ and wildtype FVB mice and C57BL6 mice as a positive control were vaccinated intramuscularly three times with hCRT-mE6mE7mL2 DNA at weekly intervals and the splenocytes harvested one week later. In the splenocytes from C57BL6 mice, robust interferon γ production by CD8+ T cells was evident in response to E6 peptides 17–19 (which encompass the known KNIVFVTVR epitope) but not E7 (**Fig 9B and 9C**). By contrast, in the splenocytes from vaccinated wildtype FVB mice, interferon-γ production by a very low percentage of CD8+ T cells was seen suggesting a small fraction of CD8+ T cells recognized E7 peptides 18–20, with no detectable response to E6 peptides (**Figs 9D, 9E, and S10–15**). A similar pattern was seen in the splenocytes from vaccinated *Tmc6*^-/-^ and *Tmc8*^-/-^ FVB mice (**S10–15 Figs**). These findings suggest that wildtype FVB mice may recognize CD8+ T cell epitopes outside of E6/E7 and/or CD4 T cell epitopes to be identified to mediate viral control.

## Discussion

Persistence of MmuPV1 after challenge of *Tmc6*^-/-^ or *Tmc8*^-/-^ mice as compared to wildtype FVB mice has many features reminiscent of typical EV in patients carrying homozygous loss of TMC6, TMC8 or CIB1 and infected with βHPV. However, there are also some differences. From the virus perspective, MmuPV1 lacks E5 and its E6/E7 coopts similar pathways to βHPV (as compared to αHPV), but it is a member of genus π, not β [51]. MmuPV1 infects cutaneous and genital sites of mice. Although genital infection with βHPVs has been reported [18–20], they typically considered skin types [52]. A recent study described a case of aggressive hrHPV+ anogenital neoplasia in a patient with typical EV and posited that their compromised ability to control cutaneous HPV may also extend to mucosal hrHPV too [53].

Uberoi *et al* first described that cutaneous papillomas developed and persisted on wildtype FVB mice after challenge with 10^8^ vge MmuPV1, but only after immunosuppression by UV exposure [38]. More recently, Torres *et al* found that challenge of *Tmc8*^-/-^ or wildtype FVB mice with 10^8^ vge MmuPV1 produced similar high burdens of skin disease, either with or without immunosuppression by UV exposure [45], arguing that MmuPv1 challenge of *Tmc8*^-/-^ mice is not a model of typical EV. Our data herein is more consistent with the first study [38] in which MmuPV1 is typically cleared by wildtype FVB mice in the absence of UV immunosuppression. However, our study is consistent with the second study in that *Tmc8*^-/-^ FVB mice developed persistent disease after MmuPV1 challenge without the use of UV immunosuppression [45].

Typical EV patients control common viral infections and generate antibody responses at, or near the normal range [17, 54]. Likewise, upon percutaneous vaccinia virus challenge, both *Tmc6*^-/-^ and *Tmc8*^-/-^ mice controlled infection and induced vaccinia neutralizing antibody titers similarly to wildtype mice. Typical EV patients exhibit plane warts associated with βHPV widely disseminated across their skin and virion-specific antibody responses. By contrast, these βHPV infections are subclinical in healthy individuals but do induce capsid-specific antibody. Both *Tmc6*^-/-^ and *Tmc8*^-/-^ mice generate antibody responses to L1/L2 VLP to similar levels as for wildtype mice [55]. Lesions were detectable in the *Tmc6*^-/-^ and *Tmc8*^-/-^ mice, but not in the wildtype FVB mice after vaginal challenge as the latter had controlled or eliminated their MmuPV1 infections at the time of sampling (**S2 Fig**). The levels of viral E6/E7 mRNA and E7, L1/L2 protein expression in the lesions detectable in the *Tmc6*^-/-^ and *Tmc8*^-/-^ mice (**S2 Fig**) were substantially lower than observed in nude mice (**S1 Fig**), suggesting some degree of repression of viral transcription by the adaptive immune system.

The levels of viral transcripts in *Tmc6*^-/-^, *Tmc8*^-/-^, wildtype and nude mice were not significantly different at 16 days post challenge (**Fig 1A**), just prior to the development of a full adaptive immune response. Similarly, infection of *Tmc6*^-/-^, *Tmc8*^-/-^, and wildtype murine keratinocytes produced similar levels of viral transcripts (**S1 Table**). These observations argue against the concept that typical EV results from the loss of a cell intrinsic immunity/intracellular restriction factor [17].

A variety of immune phenotypes have been described in typical EV patients, including reduced responses to DNCB sensitization and T cell proliferation in response to non-specific mitogens [54, 56–59], increased NK cell activity [60], normal antibody-dependent cellular cytotoxicity [61], no association of EV with particular HLA antigens [62], decreased numbers of CD4+ T cells and CD8+ T cells, but normal T suppressor cell numbers [61]. Numbers and antigen presentation function of Langerhans cells were reported as normal in EV patients, but T cells of patients with EV failed to respond to autologous epidermal cells infected with HPV [63]. This may reflect a reduction in Langerhans cells within warts because the virus suppresses CCL20 expression [64]. Supernatant of cultured PBMC obtained from EV patients exhibited depressed proliferative and IL2 responses of T cells to PHA [65]. In contrast, other EV patients exhibited normal CD4+ T cell and CD8+ T cell counts, and normal proliferative capacity in response to anti-CD3 antibody stimulation [48]. Recent studies support numbers of circulating T cells, B cells and NK cells in the normal range, and no frequency abnormalities in skin-homing T cell populations (CLA+, CCR10+, CLA+CCR4+, and CLA+CCR10+ subsets) [17]. This complex and somewhat contradictory literature likely reflects the limited number of EV patients available for study and the natural variation in human immunophenotypes, as well as the complexity and variability intrinsic to traditional assays of cell-mediated immunity. In this regard, study of the immunophenotype in *Tmc6*^-/-^ and *Tmc8*^-/-^ (and *Cib1*^-/-^) versus wildtype mice is likely to be informative.

Comparisons of *Tmc6*^-/-^, *Tmc8*^-/-^, and wildtype mice provide an opportunity to explore even subtle differences in their immune phenotype and the explore the functions of these proteins. Our studies in mice are consistent with the reported generally intact nature of the immune system in typical EV, but we observe reduced numbers of CD8+ T cells systemically and locally, and increased numbers of regulatory T cells. These differences may account for the increased persistence of MmuPV1 in *Tmc6*^-/-^ or *Tmc8*^-/-^ versus wildtype mice. If this is also the explanation for the phenotype of patients with typical EV, it is not clear why they are particularly susceptible to βHPV but not other viral pathogens. A possible reason is that control of βHPV is exclusively mediated by cellular immunity, whereas humoral immunity contributes significantly to control of most other common viral pathogens. No obvious difference in CD8+ T cell activation, proliferation or cytokine release was noted in *Tmc6*^-/-^ or *Tmc8*^-/-^ versus wildtype mice upon activation with PMA and ionomycin. Interestingly, a reduction in CD8+ T cell proliferation but not CD25 surface expression or cytokine release was noted in both *Tmc6*^-/-^ or *Tmc8*^-/-^ versus wildtype mice upon activation with anti-CD3 and anti-CD28 antibody stimulation. No deficit in surface MHC expression or antigen-presenting cells was noted either systemically or locally, in the presence or absence of MmuPV1.

Little is known about the function of Tmc6 and Tmc8 beyond their transmembrane channel-like structure, localization in calnexin positive ER-like structures [16] and binding therein with Cib1 [21]. Expression of Tmc6 and Tmc8 is robust in lymphocytes, but Tmc6 is very low and Tmc8 undetectable in keratinocytes of mice [21]. RNA data from the human protein atlas (https://www.proteinatlas.org/ version 24.0, 2024-10-22) suggests the expression of *TMC6* transcript is 10-fold higher in T cells, NK and monocytes than in keratinocytes. This difference is even more dramatic for *TMC8*, while *CIB1* transcript expression is more evenly expressed. These analyses await validation with high quality antibodies suitable for analysis of human tissues.

Tmc6 and Tmc8 bind with the zinc transporter ZnT-1, and down regulate certain Zn signaling related transcription factors (e.g. MTF-1), an activity that is blocked by HPV16 E5 [66]. Activation of CD8+ or CD4+ T cells with both anti-CD3 and CD28 antibodies results in a precipitous drop in *Tmc6* and *Tmc8* mRNA levels that is maintained over 75 h [46]. It is accompanied by increases in *ZnT1* and *ZIP10* mRNA encoding zinc transporters in a few hours and metalothionin-2 *MT2* mRNA. At 24 h post stimulation, the total free zinc levels in CD8+ or CD4+ T cells rose from ~100 nM to 400–500 nM [46]. We also observed a similar baseline and increase in cellular zinc concentration in wildtype as well as *Tmc6*^-/-^ or *Tmc8*^-/-^ CD8+ T cells at 24 h after activation (**Fig 6E**). While Lazarczyk et al observed slightly higher free zinc concentrations in EBV-transformed lymphoblastoid cell lines of EV patients compared to normal donors [46], there was no significant difference in the levels in *Tmc6*^-/-^, *Tmc8*^-/-^ or wildtype mouse-derived CD8+ T cells. This suggests that differences in zinc metabolism are not responsible for the reduced control of MmuPV1 by *Tmc6*^-/-^ or *Tmc8*^-/-^ versus wildtype mice, and that further study of TMC6, TMC8 and CIB1 function is needed.

UV exposure induces a high mutational burden [9], creating many neoantigens that could result in immune recognition and tumor control. The high rates of CSCC in atypical EV presumably reflect loss of such immune control. The earlier and increased burden of CSCC in sun-exposed areas in typical EV may occur by a similar mechanism given the lower CD8 T cell frequency and evidence of reduced activation, as well as increased regulatory T cell frequency observed the *Tmc6*^-/-^ or *Tmc8*^-/-^ versus wildtype mice, and in some patients. In this scenario, βHPV could persist and replicate at higher levels due to partial deficits in cellular immune function in classic EV. Other viral infections, such as vaccinia, whose control also involves humoral immunity could still be cleared by *Tmc6*^-/-^ or *Tmc8*^-/-^ hosts given their normal humoral and partially functional cellular immune systems, as seen in classic EV patients. If a T cell deficit underlies the ineffective clearance of MmuPV1 by *Tmc6*^-/-^ and *Tmc8*^-/-^ mice as compared to wildtype FVB, it would be expected that adoptive transfer of splenocytes from the latter would restore viral control by the knockout animals. While this was not observed (**Fig 8**), it is possible that the wildtype splenocytes transferred were insufficient in number, or not provided sufficient time (only 1 month), to control the persistent disease. Tissue-specific knockouts of *Tmc6* and *Tmc8* mice may be a more suitable method to address this question.

**Table:** 

Reagent or Resource	Source	Identifier
Antibodies		
Brilliant Violet 421 anti-mouse CD3 Antibody	BioLegend	Cat#100228
Brilliant Violet 650 anti-mouse CD8a Antibody	BioLegend	Cat#100742
**Brilliant Violet 785 anti-mouse CD4 Antibody**	BioLegend	Cat#100551
APC/Cyanine7 anti-mouse CD45 Recombinant Antibody	BioLegend	Cat#157617
PE anti-mouse CD8a Antibody	BioLegend	Cat#100707
BD OptiBuild BV421 Mouse Anti-Mouse H-2D[q]/H-2L[q]	BD OptiBuild	Cat#744853
BD Pharmingen Alexa Fluor 647 Rat Anti-Mouse I-A/I-E	BD Pharmingen	Cat#562367
FITC anti-human/mouse CD49f Antibody	BioLegend	Cat#313605
PE/Dazzle 594 anti-mouse CD11c Antibody	BioLegend	Cat#117347
Zombie Aqua Fixable Viability Kit	BioLegend	Cat#423101
PE anti-mouse CD19 Antibody	BioLegend	Cat#152407
APC/Fire 750 anti-mouse CD3 Antibody	BioLegend	Cat#100247
PE anti-mouse CD3 Antibody	BioLegend	Cat#100205
Brilliant Violet 421 anti-mouse FOXP3 Antibody	BioLegend	Cat#126419
APC/Fire 750 anti-mouse CD25 Antibody	BioLegend	Cat#102053
APC anti-mouse IFN-γ Antibody	BioLegend	Cat#505810
Reagents		
EasySep Mouse Naïve CD8+ T Cell Isolation Kit	Stem Cell Technologies	Cat# 19858
CellTrace Far Red Cell Proliferation Kit	Invitrogen	Cat#C34564
FluoZin-3, AM, cell permeant	Invitrogen	Cat# F24195
N,N,N′,N′ Tetrakis(2pyridylmethyl)ethylenediamine	Sigma-Aldrich	Cat# P4413
Zinc sulfate heptahydrate	Sigma-Aldrich	Cat# Z0251
2-Mercaptopyridine N-oxide sodium salt	Sigma-Aldrich	Cat# H3261
BD Pharmingen Purified Rat Anti-Mouse CD16/CD32 (Mouse BD Fc Block)	BD Pharmingen	Cat#553142
eBioscience Intracellular Fixation & Permeabilization Buffer Set	Invitrogen	Cat#88-8824-00
Ultra-LEAF Purified anti-mouse CD3 Antibody	BioLegend	Cat#100238
Ultra-LEAF Purified anti-mouse CD28 Antibody	BioLegend	Cat#102116
Cell Activation Cocktail (with Brefeldin A)	BioLegend	Cat#423303
Cell Activation Cocktail (without Brefeldin A)	BioLegend	Cat# 423301
Brefeldin A Solution (1,000X)	BioLegend	Cat#420601
Glass beads	Sigma Aldrich	G4649
Quick-DNA Universal Kit	Zymo Research	D4068
Trizol Reagent	Thermo Fisher	15596026
RNeasy Mini Kit	Qiagen	74104
QuantiTect Reverse Transcription Kit	Qiagen	205310
TaqMan Gene Expression Master Mix	Thermo Fisher	4369016

## Methods

### Mice and ethics statement

All animal studies were carried out in accordance with the recommendations in the Guide for the Care and Use of Laboratory Animals and with the prior approval of the Animal Care and Use Committee of The Johns Hopkins University (protocol MO21M125, MO24M243). Four- to five-week-old male and female FVB mice (Taconic) and athymic nude mice [Crl:NU(NCr)-Foxn1nu, obtained from Charles River Laboratories). *Tmc6*^-/-^ (*Ever1*^-/-^) and *Tmc8*^-/-^ (*Ever2*^-/-^) mice were generated on a 129 background, bred, and maintained on the C57BL/6 Cre inbred genetic background in the homozygous state [21], and backcrossed to an FVB/N inbred genetic background as previously described [45]. The *Tmc6*^-/-^ and *Tmc8*^-/-^ mice were bred from colonies established at Johns Hopkins University and were genotyped prior to use. FVB/N (Taconic) mice were purchased to provide non-transgenic, syngeneic mice as wild-type controls for our studies.

### Biostatistics

Two-sample t-tests and paired t-tests were applied to compare continuous outcomes between independent groups and paired samples, respectively. For comparisons involving multiple groups, one-way ANOVA was utilized to assess significant differences among group means. When ANOVA results indicated significant differences, post-hoc pairwise comparisons were performed using two-sample t-tests, with p-values adjusted for multiple comparisons according to the Benjamini-Hochberg (BH) method [67]. All p-values were two-sided, with a significance threshold set at 0.05. Statistical analyses were conducted using Prism 10 and R (Version 4.2.2).

### Preparation and titering of MmuPV1 wart lysate

As previously described, athymic nude mice were used to propagate MmuPV1 viruses [32]. For MmuPV1 wart lysate used in the vaginal and tail challenges, frozen tail warts were thawed and then homogenized with glass beads (G4649, Sigma/Aldrich) in DPBS containing 0.8M NaCl by using a minibeadbeater (BioSpec Products). The wart extract was centrifuged to remove tissue debris, and the supernatant was collected and stored at -20°C until use. For the flow cytometry experiments, wart tissue was homogenized in MACS Dissociation M tube (130-096-335, Miltenyi Biotech), using a GentleMACS Dissociator (setting Protein_0.10.1). To determine the MmuPV1 titer, DNA was extracted from 200 μL of wart lysate using Quick-DNA Universal Kit (D4068, Zymo Research) and amplified in triplicate using TaqMan Gene Expression Master Mix in a qPCR reaction containing forward primer 5’-AGAGTGCATGGCTGGCAAGA-3’ (nucleotides 255–274 of NC_014326.1), reverse primer 5’-CATGTGGCGCACCAAGTGAA-3’ (nucleotides 384–365 of NC_014326.1) and probe 5’FAM-TGGCAAGCCGCACGCTTTGGCATCA-3’TAMRA (nucleotides 363–339 of NC_014326.1) within the E6 region with a cycling program of 2 min at 50°C, 10 min at 95°C and 40 cycles of 15 sec for 95°C, 1 min at 60°C. For quantitation standard, a plasmid containing sequences of the MmuPV1 genome was used. The stock plasmid was diluted 100-fold serially from 10^0^ to 10^10^ vge and amplified with the wart lysate DNA samples. No MmuPV1 DNA was detected in no-template control.

### Tail challenge with MmuPV1

MmuPV1 wart lysate was diluted 1:1 with PBS. Challenge of the tail was preceded by using 2 to 5 passes of a Dremel rotary drill (setting 2) until the epidermis was disturbed in a 2 cm long region. Then, 20 μL of MmuPV1 wart lysate containing half of the stated challenge dose was applied onto the tail, and spread using an endocervical brush (892010-C500, Andwin Scientific). An additional 20 μL of MmuPV1 lysate containing the second half of the stated challenge dose was applied after brushing.

### Vaginal challenge with MmuPV1

Mice were injected subcutaneously with 3 mg of medroxyprogesterone (Depo-Provera, NDC59762-4537-1) 4 days before infection challenge to synchronize their estrous cycles. For infection of the vaginal tract, 20 μL of MmuPV1 wart lysate/3%CMC in PBS (1:1 *v*/*v*) were introduced to the vaginal vault of anesthetized mice (80 mg/kg ketamine and 8 mg/kg xylazine) before (containing half of the stated challenge dose) and 20 μL after abrasion (containing the second half of the stated challenge dose) by twirling an endocervical brush clockwise and anti-clockwise 10 times each.

### RNA extraction from vaginal and tail tissues

Vaginal tract and tail tissues were dissected immediately after euthanasia, and homogenized in 0.3 ml of Trizol in MACS Dissociation M tubes (130-096-335, Miltenyi Biotech) using the gentleMACS Dissociator (setting RNA_02.01). After homogenization, the M tube was centrifuged at 4000 rpm for 5 min at 4°C. The supernatant was transferred to a new 1.5 mL Ependorf tube and ~0.6 mL of Trizol was added to make the total volume up to 1 mL. 200 μL of chloroform:isoamyl alcohol, 24:1 (C0549-1PT, Sigma/Aldrich) was added per 1.0 mL of Trizol used. The tube was shaken vigorously for 15 sec and incubated at room temp for 2–3 min before spinning at 12,000 x *g* for 15 min at 4°C in a refrigerated microfuge. The top aqueous layer was transferred to a new tube. The new tube was centrifuged as before, and the aqueous layer was transferred to another new tube. One volume of 70% ethanol was added to the aqueous phase and RNA was extracted using Qiagen RNeasy Mini Kit.

### Vaginal sampling with a cytobrush

Mice were anesthetized (80 mg/kg ketamine and 8 mg/kg xylazine) and an endocervical brush soaked in 1xPBS was inserted and twirled inside the vaginal vault several times and dipped in 0.3 mL of Trizol. The samples were stored at -20°C until use.

### RNA extraction from vaginal sampling

Vaginal scrapings were thawed on ice and homogenized by hand with plastic homogenizer sticks (47747–366, VWR) for 20 strokes. After homogenization, ~0.6 mL of Trizol was added to make up to 1 mL. The sample was incubated at room temp for 5 min to permit complete dissociation of the nucleoprotein complex. 120 μL of chloroform:isoamyl alcohol, 24:1 (C0549-1PT, Sigma/Aldrich) was added per 0.3 mL of Trizol used. The tube was shaken vigorously for 15 sec and incubated at room temp for 2–3 min before spinning at 12,000 x *g* for 15 min at 4°C in a refrigerated microfuge. The top aqueous layer was transferred to a new tube. The new tube was spun as above. The aqueous layer was transferred to another new tube. 150 μL of isopropanol (BP2618-500, Fisher Scientific) was added per 1 mL of Trizol used. The mixture was incubated for 10 min at room temp before spinning at 12,000 x *g* for 10 min at 4°C. The supernatant was discarded and the pellet of RNA was kept. 1 ml of 75% ethanol (absolute ethanol, BP2818-500, Fisher Scientific) was added to the pellet and mixed by shaking before spinning at 7500g for 5 min at 4°C. The pellet was air-dried for 5–10 min and resuspended in 30 μL of ultrapure water (351-029-131, Quality Biological) and concentration was determined using 2 μL of the RNA solution using a Nano drop.

### qPCR for MmuPV1 and CD8a mRNA

cDNA synthesis was performed using QuantiTect Reverse Transcription Kit. For detection of MmuPV1 expression, sequences for forward, reverse primers and probe are as follows: 5’- TAGCTTTGTCTGCCCGCACT-3’ (nucleotides 695–714 of NC_014326.1), 5’- GTCAGTGGTGTCGGTGGGAA-3’ (nucleotides 3232–3213 of NC_014326.1), 5’FAM-CGGCCCGAAGACAACACCGCCACG-3’TAMRA (nucleotides 3179–3202 and of NC_014326.1). A plasmid with the 157 bp MmuPV1 amplicon cloned into the EcoRV site in pUC57 was used as positive control. For detection of CD8a expression, TaqMan Assay for mouse *Cd8a* (FAM-labeled assay, Mm01182108_m1, Thermo Fisher) was used. Capping Actin Protein Of Muscle Z-Line Subunit Beta (Capzb) was used as the reference housekeeping gene (VIC-labeled assay, Mm00486707_m1, Thermo Fisher). Multiplex real-time PCR assays were set up in triplicate using TaqMan Gene Expression Master Mix, with a cycling program of 2 min at 50°C, 10 min at 95°C and 40 cycles of 15 sec at 95°C, 1 min at 60°C. No MmuPV1 or *Cd8a* transcripts were detected in the absence of either template or reverse transcriptase. Statistical analysis was performed using Student’s T test.

### Culture and infection of keratinocytes from adult mouse tails with MmuPV1

MmuPV1 wart lysate at 1.86x10^9^ vge/μL was prepared by homogenization with GentleMACS Dissociator (130-093-235, Miltenyi Biotech). For infection of primary keratinocytes, the extracellular matrix (ECM)-to-cell transfer method of Sapp et al [68] was modified as follows. HaCaT cells were seeded at 3x10^5^ cells/well in 6-well plates. The cells were removed by incubating in 0.5mM EDTA (15575–038, Invitrogen) in 1xDPBS (114-057-101, Quality Biologicals) for 2 h. After rinsing with 1xPBS (10010–023, Gibco), the 6-well plates containing extracellular matrix were stored at -20°C until use. On the day of infection, the plates were thawed and rinsed with 1xPBS, MmuPV1 wart lysate in keratinocyte culture medium was added and incubated for 1 h before addition of keratinocytes. The method for culturing primary keratinocytes was adapted from [69]. 2–3 months old *Tmc6*-/-, *Tmc8*-/- and wildtype FVB mice were euthanized and their detached tails were washed in 70% ethanol, transferred to a 100mm dish and rinsed in 1xPBS. A disposal scalpel was used to cut along the tail after the tail tip was cut off with a razor blade. The skin was peeled off the bone and cut into 4–5 pieces 1–2 cm long. The tail pieces were incubated with gentle rotation at 4°C overnight, in a 15 mL tube containing 10 mL of DispaseII (17105–041, Gibco) at 2.4U/mL. The next day, the tail pieces were transferred to a 100 mm dish and rinsed in 1XPBS. The epidermal layer was peeled off from the dermal layer. Trypsin-EDTA (25200056, Gibco) was added onto the epidermal layer and incubated at 37°C for 10–15 min. DMEM (11965118, Gibco) containing 10%FBS (900–108, Gemini Bio-Products) was added to the trypsinized epidermis. Cells were then shaken off with forceps and cell clumps were broken up by tituration. After passing through a 100 μm cell strainer (352360, Fisher Scientific), cells were centrifuged for 5 min at 250*g* at room temperature. The cell pellet was resuspended in keratinocyte culture medium (37010022, Gibco) containing 0.05mM CaCl_2_ (351-130-721, Quality Biologicals), 50U/mL penicillin-50μg/mL streptomycin (15140122, Gibco) and supplemented with 5ng/mL human recombinant epidermal growth factor (10450–013, Gibco) and 50 μg/mL bovine pituitary extract (130-28-014, Gibco). Cells were counted and seeded at 3x10^5^ cells/well in 6-well plates containing extracellular matrix from HaCaT cells and various doses of MmuPV1 wart lysate. The cells were incubated with MmuPV1 for 72 h and harvested by incubating in Trypsin-EDTA for 10–15 min. DMEM+10%FBS was added and the cells were centrifuged at 250*g* for 4 min at room temperature. The cell pellet was rinsed with 1xPBS and centrifuged again before adding 1 ml of Trizol. Samples were stored at -20°C until RNA extraction and qPCR for MmuPV1 RNA.

### Infectivity of VV-luc in mice

6–8 weeks of old wild-type FVB/NTac, or Tmc6^-/-^, or Tmc8^-/-^ FVB mice were anesthetized (60 mg/kg ketamine and 8 mg/kg xylazine) and 5x10^5^ pfu (5 μL) of VV-luc was applied to the dorsal surface of the tail, ~1 cm below its base. The skin area was then gently scarified 15 times with a bifurcated needle. Mice were imaged by IVIS Spectrum in vivo imaging system series 2000 (PerkinElmer) while under anesthesia with 3% isoflurane inhalation at indicated time points after i.p. injection of luciferin. Total photon counts were quantified in the challenge site by using Living Image 2.50 software (PerkinElmer).

### Measurement of vaccinia neutralizing antibodies

To test neutralization ability of sera from vaccinated mice (n = 5), 1x10^5^ pfu (1μL) of vaccinia virus expressing luciferase (VV-luc) was pre-mixed with serially diluted sera for 1 h at ambient temperature, then the mixture was added to 1x10^4^ 293TT cells/well of a 96-well plate. After incubation for 24 h, cells were lysed, one step Luciferase Reagent buffer (BPS Bioscience) and Luciferase Reagent substrate were added and luciferase activity measured in a luminometer. Anti-vaccinia virus antibodies Mab 10F5, 7d11 monoclonal antibodies (0.78 μg/mL) were used as positive control (kind gift of J Hooper, USAMRID), along with non-neutralizing 20-VR69 rabbit antiserum to vaccinia virus (lot P13091802, 25 μg/mL, Fitzgerald).

### Immunohistochemical staining of tissue sections

Rabbit antisera were generated against full length MmuPV1 E7 produced with a 6His tag in *E*. *coli*, and against MmuPV1 L1/L2 VLP expressed in 293TT cells and purified by density gradient centrifugation. Briefly, formalin-fixed, paraffin-embedded sections were deparaffinized in xylene, followed by dehydration in graded ethanol. Antigen retrieval was performed by steaming specimens at 100°C for 20 min in Target Retrieval Solution (Dako) and subsequently washed in Tris-buffered saline with Tween 20 (TBST, 0.05% Tween 20). Endogenous peroxidase was blocked, by treatment of slides with Dual Endogenous Enzyme-Blocking Reagent (Dako) for 5 min at room temperature. Sections were covered with rabbit polyclonal primary antibody against MmuPV1 E7 (1:1200) or L1/L2 (1:200) diluted with Antibody Dilution Buffer (ChemMate) and then incubated at room temperature for 45 min. Slides were then washed with TBST, followed by incubation with PowerVision Poly-HRP Anti-Rabbit IgG for 30 min at room temperature. After three washes in TBST, sections were treated with DAB chromogen (3, 3 ’-diaminobenzidine tetrahydrochloride; Sigma) for 20 min in the dark. Sections were counterstained with Mayer’s hematoxylin (Dako), dehydrated with ethanol and xylene, and mounted permanently.

### Chromogenic in situ hybridization

Custom RNA in situ hybridization probes were developed to detect the full-length E6/E7 mRNA sequence of MmuPV1 by Advanced Cell Diagnostics for use with their RNAscope assay kit. CISH was performed according to the manufacturer’s instructions for RNAscope2.0 as previously described [32]. Briefly, formalin-fixed, paraffin-embedded mouse tail and vaginal tissue sections were pretreated with heat and protease prior to hybridization with the probe. If treated with RNase A (Qiagen), slides were incubated for 30 min at room temperature with RNase A in PBS (10 mg/mL). Slides were subsequently washed three times in diethyl pyrocarbonate (DEPC)-treated water for 5 min per wash. To ensure RNA integrity and assay procedures, adjacent sections were also hybridized with a probe for the endogenous housekeeping gene ubiquitin. After washing, a horseradish peroxidase (HRP)-based amplification system was then used to detect the target probes, followed by color development with 3,3′-diaminobenzidine (DAB).

### In vitro assay for effector T cell function

Lymphocytes were isolated from the spleens of *Tmc6*^-/-^, *Tmc8*^-/-^, and FVB mice, with or without MmuPV1 challenge. RBCs were lysed by adding an excess amount of RBC lysis buffer (46232, Cell Signaling Technology) twice, followed by extensive washing with 0.5% BSA in PBS (FACS buffer). To evaluate the functionality of CD8+ T cells, 2x10^5^ cells were resuspended in 200 μL of RPMI supplemented with 10% FBS and T-cell stimulants: either α-CD3 (2.5 μg/mL) and α-CD28 (0.25 μg/mL) antibodies, or phorbol 12-myristate-13-acetate (PMA, 80 nm) and 1.33 μM Ionomycin (Cell Activation Cocktail; BioLegend), followed by the addition of Brefeldin A for 16 h. Subsequently, the cells were harvested for IFNγ analysis via flow cytometry.

### In vitro CD8 T cell activation and proliferation

Lymphocytes were taken from the spleens of *Tmc6*^-/-^, *Tmc8*^-/-^, and wildtype FVB mice. RBCs were lysed by the addition of excess RBC lysis buffer twice (46232, Cell Signaling Technology) followed by extensive washing in 0.5% BSA/PBS (FACS buffer). Naïve CD8 T cells were magnetically enriched using the EasySep Mouse Naïve CD8+ T Cell Isolation Kit according to manufacturer instructions. The isolation efficiency of naïve CD8 T cells was confirmed by flow cytometry analysis. For the CD8 T cell proliferation assay, lymphocytes (1x10^6^ cells/mL) were suspended in PBS and labeled with the CellTrace Far Red at 10 μM at 37°C for 20 min. After labeling, an excess of 10% RPMI/FBS was added to the samples to quench the reaction. Following centrifugation and extensive washing, cells were resuspended in 200 mL of 10% RPMI/FBS with T cell stimulant: either anti-CD3 (2.5 μg/mL) and anti-CD28 (0.5 μg/mL) antibodies or Phorbol 12-myristate 13-acetate (80 nM)/Ionomycin (1.33 μM) for 48 h. Cells were then harvested for analysis by flow cytometry.

### Flow cytometry

Lymphocytes harvested from spleens were processed into single-cell suspensions. Red blood cells were twice lysed by adding a 20-fold volume of 1X RBC lysis buffer (46232, Cell Signaling Technology) and collected by centrifugation, and then washed in 0.5% BSA/PBS (FACS buffer). Single staining controls using UltraComp eBeads (01-2222-42, Thermo Fisher Scientific) were employed to establish a compensation matrix for each experiment. Fluorescence minus one (FMO) and isotype staining controls were utilized to set gating parameters and assess non-specific binding. Prior to antibody staining, Zombie Aqua Fixable Viability kit was applied for dead cell discrimination, following Biolegend’s instructions. Mouse Fc Block anti-mouse CD16/CD32 mAb 2.4G2 (≤ 1 μg/million cells in 100 μL) at 4°C for 5 minutes was added before antibody staining to prevent nonspecific binding. Antibody staining was conducted for at least 20 min at 4°C. For the measurement of IFNγ cytokine production through intracellular staining, mouse lymphocytes were incubated with 5 μg/mL Brefeldin A for 16 h. Subsequently, cells were processed as outlined in the flow cytometry protocol. Following extracellular staining, cells were permeabilized using the eBioscience Foxp3/Transcription Factor Staining Buffer Set (00-5523-00, Thermo Fisher Scientific) and stained for intracellular cytokines following Thermo’s “Protocol B: One-step protocol: intracellular (nuclear) proteins” in a 96-well plate. After the final wash, the supernatant was discarded and the sample pellet completely dissociated by pulsed vortexing.

200 μL of Foxp3 Fixation/Permeabilization working solution was added to each well such that the cells are fully resuspended in the solution. The cells were incubated for 30 minutes at room temperature away from light and collected by centrifugation at 500 x *g* for 5 min. After discarding the supernatant, 200 μL 1X Permeabilization Buffer was added to each well and the cells collected by centrifugation at 500 x *g* for 5 min at room temperature; this was repeated.

The cell pellet was resuspended in a total 100 μL of 1X Permeabilization Buffer with 0.04% normal mouse serum. After 15 min at room temperature, APC anti-mouse IFN-γ antibody was added for 30 minutes at room temperature while protected from light. 200 μL of 1X Permeabilization Buffer was added to each well and the cells harvested by centrifugation at 500 x g for 5 min, the supernatant discarded and the wash repeated. Finally the stained cells were resuspended in Flow Cytometry Staining Buffer for flow cytometry analysis. Data acquisition was performed on a 13-color Beckman Coulter CytoFLEX S, and analysis was conducted using FlowJo software version 10.4 (FlowJo LLC).

### Cellular Zinc concentration measurement

Magnetically enriched naïve CD8 T cells derived from the spleens of *Tmc6*^-/-^, *Tmc8*^-/-^, and wildtype FVB mice were resuspended in 200 mL of 10% RPMI/FBS with T cell stimulant: either α-CD3 (5 μg/mL) and α-CD28 (1 μg/mL) antibodies or PMA/Ionomycin in a 96-well plate for 24 h. Cells were harvested and aliquoted, each in three wells of a V-shaped 96-well plate. The measurement of cellular zinc concentration in lymphocytes by flow cytometry was previously described [70]. Briefly, the cells were loaded with FluoZin-3 AM ester (1 mM) in PBS for 30 minutes at room temperature in the dark. Each experiment contained 3 samples: the “minimum” control, the “maximum” control, and the experimental sample. The minimum control was determined by the addition of the zinc-specific, membrane-permeant chelator TPEN (100 mM), and the maximum was determined by the addition of ZnSO_4_ (100 mM) and the ionophore pyrithione (50 mM). The concentration of intracellular labile zinc was calculated from the mean fluorescence of FluoZin-3 AM with the formula [Zn] = K_d_.((F-Fmin)/(Fmax-F)). The dissociation constant of the FluoZin-3 AM is 15 nM [71].

## Supporting information

S1 Fig Histologic analyses of MmuPV1-challenged nude mice for morphology, early transcript and MmuPV1 E7 or L1/L2 protein expression. The vaginal tissue and tail samples from a nude mouse challenged with MmuPV1 on the tail and intravaginally were fixed in formalin at 2 months post challenge, paraffin-embedded and sectioned. A section of tail from the nude mouse was stained with (**A**) H&E, (**B**) RNAscope to detect MmuPV1 transcript, (**C**) Immunohistochemistry to detect MmuPV1 E7, or (**D**) MmuPV1 L1/L2 VLP. A section of vaginal tract from the nude mouse was stained with (**E**) H&E, (**F**) RNAscope to detect MmuPV1 transcript, (**G**) Immunohistochemistry to detect MmuPV1 E7, or (**H**) MmuPV1 L1/L2 VLP.
(TIF)

S2 Fig Histologic analyses of MmuPV1-challenged wildtype, *Tmc6*^-/-^ and *Tmc8*^-/-^ FVB mice for morphology, early transcript and MmuPV1 E7 or L1/L2 protein expression. The vaginal tissue from the mice described in **Fig 1A–1F** were fixed in formalin at 2 months post challenge with MmuPV1, paraffin-embedded and sectioned. A representative section of vaginal tract from a wildtype FVB mouse was stained with (**A**) H&E, (**B**) RNAscope to detect MmuPV1 transcript, although no signal was observed consistent with the RT-PCR data. A representative section of vaginal tract from a *Tmc6*^-/-^ mouse was stained with (**C**) H&E, or (**D**) RNAscope to detect MmuPV1 transcript. A representative section of vaginal tract from a *Tmc8*^-/-^ mouse was stained with (**E**) H&E, or (**F**) RNAscope to detect MmuPV1 transcript.
(TIF)

S3 Fig RT-PCR analysis of MmuPV1 and *Cd8a* transcripts in vaginal swab samples. Wildtype, *Tmc6*^-/-^ and *Tmc8*^-/-^ mice (n = 5/group, 7–10 week old females) were challenged intra-vaginally with 10^8^ vge of MmuPV1 and compared to mock-challenged mice (**Fig 2**). A vaginal sample was obtained at 17 days post infection (see **Fig 3**), and the mice euthanized at week 3. The success of the challenge was confirmed by measuring MmuPV1 early transcript as compared to *Capzb* housekeeping gene transcript levels in vaginal tissue collected at 3 weeks (**A**), or *Cd8a* transcript levels (**B**) by RT-PCR. (**C**) As described in **Fig 4**, Wildtype, *Tmc6*^-/-^, and *Tmc8*^-/-^ mice (10–14 week old females) were challenged with 3.7x10^10^ vge MmuPV1(n = 5/group) or naïve (n  =  2 wildtype and 3 *Tmc6*^-/-^). Samples where no signal was detected at 40 cycles were assigned a value of 40 (indicated with red dot).
(TIF)

S4 Fig Representative gating of IFN*γ*+ CD8+ T cells in Fig 5E. (TIF)

S5 Fig Representative gating of IFN*γ*+ CD8+ T cells in Fig 5F. (TIF)

S6 Fig Representative gating of CD25+ CD8+ T cells in Fig 6C. (TIF)

S7 Fig Representative gating of dividing CD8+ T cells in Fig 6D. (TIF)

S8 Fig Imaging of infection by vaccinia virus expressing luciferase. (**A**) Schematic illustration of the experiment. Briefly, 6–8 week old wild-type FVB, *Tmc6*^-/-^, or *Tmc8*^-/-^ male mice (n = 5/group) anesthetized and 5x10^5^ pfu (5 μL) of vaccinia virus expressing luciferase was applied to tail skin, 1 cm from the base of the tail. The skin area was then gently scratched 15 times with a bifurcated needle. Mice were imaged by IVIS Spectrum in vivo imaging system series 2000 (PerkinElmer) at days 1, 6, 10 and 14. Total photon counts were quantified in the tumor site by using Living Image 2.50 software (PerkinElmer). (**B**) Representative luminescence imaging from each group. (**C**) Quantification of luminescence signal in interest area.
(TIF)

S9 Fig Measurement of serum neutralizing antibody response to vaccinia virus. In vitro measurement of neutralizing serum antibody titer generated in response to percutaneous vaccinia virus challenge of wildtype, *Tmc6*^-/-^ and *Tmc8*^-/-^ FVB mice. (**A**) Anti-vaccinia virus monoclonal antibodies 10F5, 7d11 were used as positive control, and non-neutralizing serum 20VR69 as a negative control. (**B**) To assay neutralization titer of sera from challenged mice, 1x10^5^ pfu of vaccinia virus expressing luciferase (VV-luc) was pre-mixed with serially diluted sera for 1 h, then the mixture was added to 1 X 10^4^ 293TT cells in the well of a 96-well plate. After incubation for 24 h, cells were lysed, BPS Bioscience one step Luciferase Reagent buffer and Luciferase Reagent substrate were both added and luciferase activity measured in a luminometer.
(TIF)

S10 Fig Measurement and mapping of CD8 T cell response of FVB mice to hCRT-mE6mE7mL2 DNA vaccination. Characterization of MmuPV1 E6 and E7 epitopes recognized by CD8+ T cells of FVB mice. Mice (n = 3) were injected intramuscularly with 15μg of hCRT-mE6mE7mL2 DNA vaccine followed by electroporation on days 1, 8 and 15. One week after the final vaccination, splenocytes were collected, as were splenocytes from unvaccinated mice. To determine the epitopes, a panel of 20mer peptides, each overlapping by 15 amino acids were incubated with splenocytes in the presence of Golgi plug for 16 hours. Splenocytes stimulated with eBioscience Cell Stimulation Cocktail was used as a positive control. The cells were stained for interferon-γ and CD8, and analyzed by flow cytometry using a CytoFLEX S (Beckman) and data were analyzed by FlowJo software. Percentage of interferon-γ producing CD8+ T cells is presented.
(TIF)

S11 Fig Measurement and mapping of CD8 T cell response of Tmc6^-/-^ and Tmc8^-/-^ mice to hCRT-mE6mE7mL2 DNA vaccination. Characterization of MmuPV1 E6 epitopes recognized by CD8+ T cells of Tmc6^-/-^ and Tmc8^-/-^ FVB mice as in **S10 Fig**.
(TIF)

S12 Fig Measurement and mapping of E7-specific CD8 T cell response of FVB mice to hCRT-mE6mE7mL2 DNA vaccination. Characterization of MmuPV1 E7 epitopes recognized by CD8+ T cells of FVB mice as in **S10 Fig**.
(TIF)

S13 Fig Measurement and mapping of E7-specific CD8 T cell response of Tmc6^-/-^ and Tmc8^-/-^ mice to hCRT-mE6mE7mL2 DNA vaccination. Characterization of MmuPV1 E7 epitopes recognized by CD8+ T cells of Tmc6^-/-^ and Tmc8^-/-^ FVB mice as in **S10 Fig**.
(TIF)

S14 Fig Measurement and mapping of E7-specific CD8 T cell response of FVB mice to hCRT-mE6mE7mL2 DNA vaccination. Characterization of MmuPV1 E7 epitopes recognized by CD8+ T cells of FVB mice as in **S10 Fig**.
(TIF)

S15 Fig Measurement and mapping of E7-specific CD8 T cell response of Tmc6^-/-^ and Tmc8^-/-^ mice to hCRT-mE6mE7mL2 DNA vaccination. Characterization of MmuPV1 E7 epitopes recognized by CD8+ T cells of Tmc6^-/-^ and Tmc8^-/-^ FVB mice as in **S10 Fig**.
(TIF)

S1 Table MmuPV1 infection of keratinocytes cultured from *Tmc6*^-/-^, *Tmc8*^-/-^ or wildtype mice. A male mouse from each genotype, (**A**) wildtype FVB, (**B**) *Tmc6*^-/-^, or (**C**) *Tmc8*^-/-^ of 2–3 months in age were sacrificed and keratinocytes cultured from the tails harvested. Keratinocytes were infected with 0.02 μL and 0.2 μL of MmuPV1 (1.86x10^9^ vge/μL), 3 wells each. The Cq for samples that did not amplify at 40 cycles were indicated as 40.
(DOCX)

S2 Table RT-PCR analysis of MmuPV1 and *Capzb* transcript levels at 2 months after tail challenge. The Cq for samples that did not amplify at 40 cycles were indicated as 40.
(DOCX)

S1 Data File Readers can click on graphs and obtain primary data in Graphpad Prism. (XLSX)
