# Supplementary figures and images for: MmuPV1 infection of Tmc6/Ever1 or Tmc8/Ever2 deficient FVB mice as a model of βHPV in typical epidermodysplasia verruciformis
Source: PLoS Pathog. 2025 Jan 15;21(1):e1012837. doi: 10.1371/journal.ppat.1012837 (PMC11734914; doi:10.1371/journal.ppat.1012837)

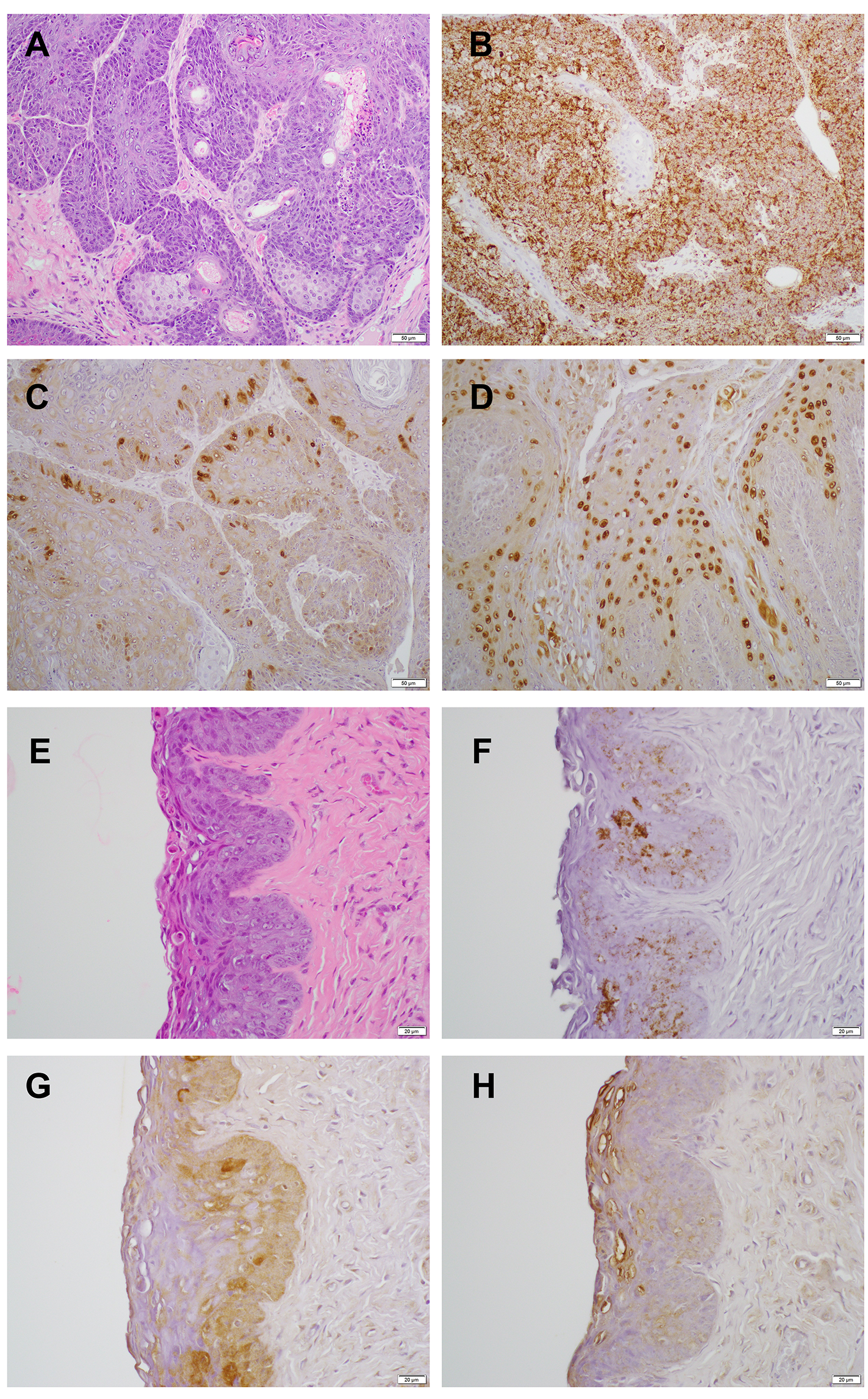

Supplement: S1 Fig — The vaginal tissue and tail samples from a nude mouse challenged with MmuPV1 on the tail and intravaginally were fixed in formalin at 2 months post challenge, paraffin-embedded and sectioned. A section of tail from the nude mouse was stained with (A) H&E, (B) RNAscope to detect MmuPV1 transcript, (C) Immunohistochemistry to detect MmuPV1 E7, or (D) MmuPV1 L1/L2 VLP. A section of vaginal tract from the nude mouse was stained with (E) H&E, (F) RNAscope to detect MmuPV1 transcript, (G) Immunohistochemistry to detect MmuPV1 E7, or (H) MmuPV1 L1/L2 VLP. (TIF) [file ppat.1012837.s001.tif]

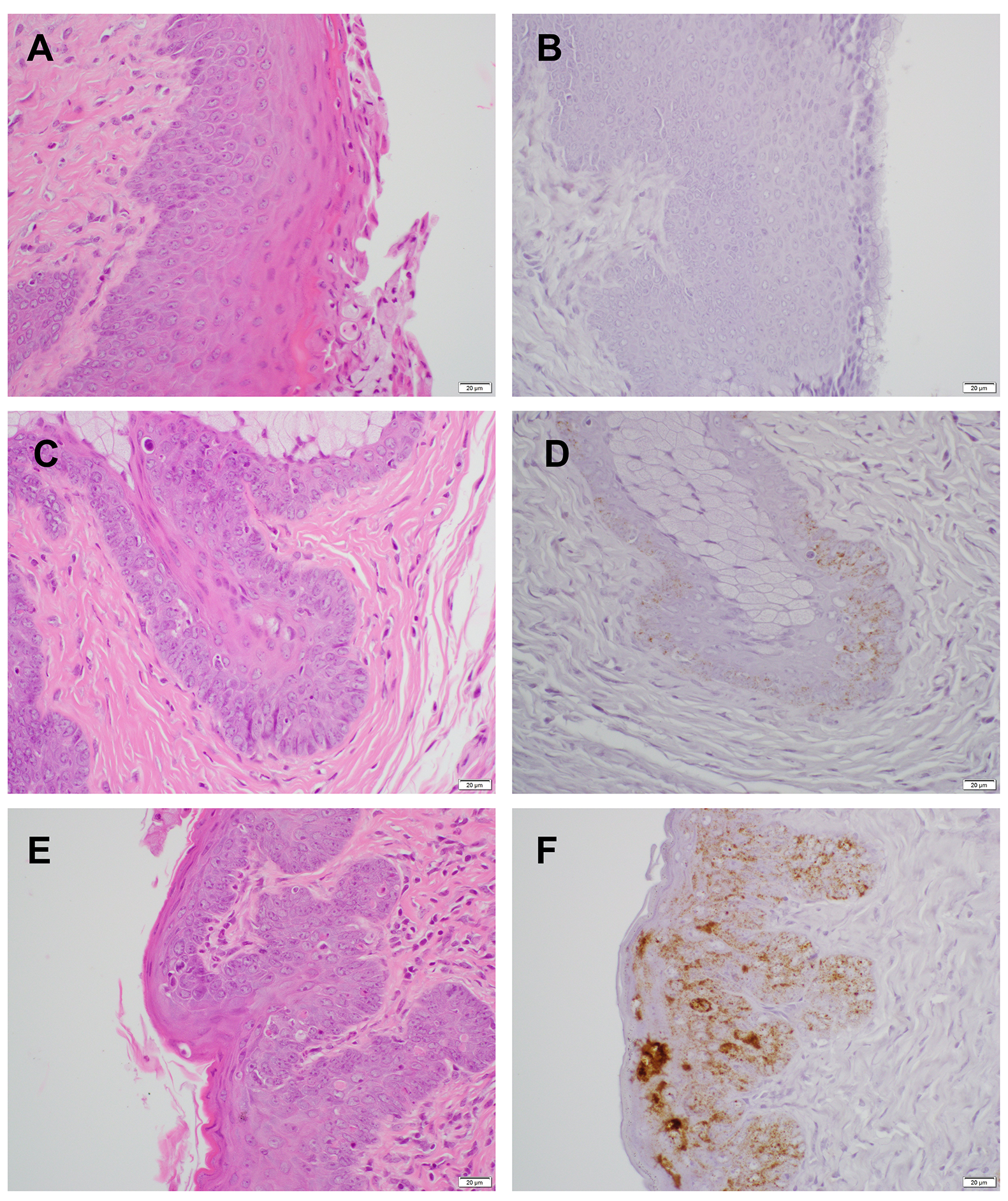

Supplement: S2 Fig — The vaginal tissue from the mice described in Fig 1A–1F were fixed in formalin at 2 months post challenge with MmuPV1, paraffin-embedded and sectioned. A representative section of vaginal tract from a wildtype FVB mouse was stained with (A) H&E, (B) RNAscope to detect MmuPV1 transcript, although no signal was observed consistent with the RT-PCR data. A representative section of vaginal tract from a Tmc6-/- mouse was stained with (C) H&E, or (D) RNAscope to detect MmuPV1 transcript. A representative section of vaginal tract from a Tmc8-/- mouse was stained with (E) H&E, or (F) RNAscope to detect MmuPV1 transcript. (TIF) [file ppat.1012837.s002.tif]

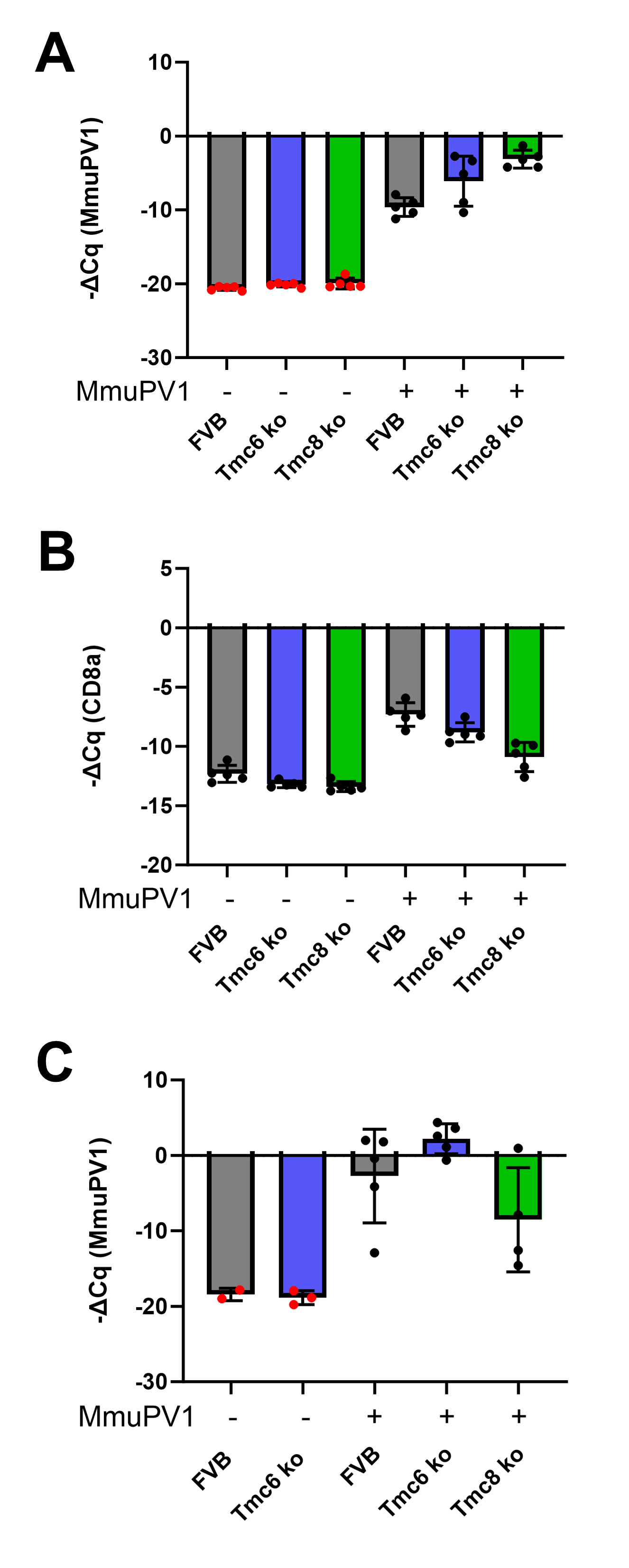

Supplement: S3 Fig — Wildtype, Tmc6-/- and Tmc8-/- mice (n = 5/group, 7–10 week old females) were challenged intra-vaginally with 108 vge of MmuPV1 and compared to mock-challenged mice (Fig 2). A vaginal sample was obtained at 17 days post infection (see Fig 3), and the mice euthanized at week 3. The success of the challenge was confirmed by measuring MmuPV1 early transcript as compared to Capzb housekeeping gene transcript levels in vaginal tissue collected at 3 weeks (A), or Cd8a transcript levels (B) by RT-PCR. (C) As described in Fig 4, Wildtype, Tmc6-/-, and Tmc8-/- mice (10–14 week old females) were challenged with 3.7x10^10^ vge MmuPV1(n = 5/group) or naïve (n = 2 wildtype and 3 Tmc6-/-). Samples where no signal was detected at 40 cycles were assigned a value of 40 (indicated with red dot). (TIF) [file ppat.1012837.s003.tif]

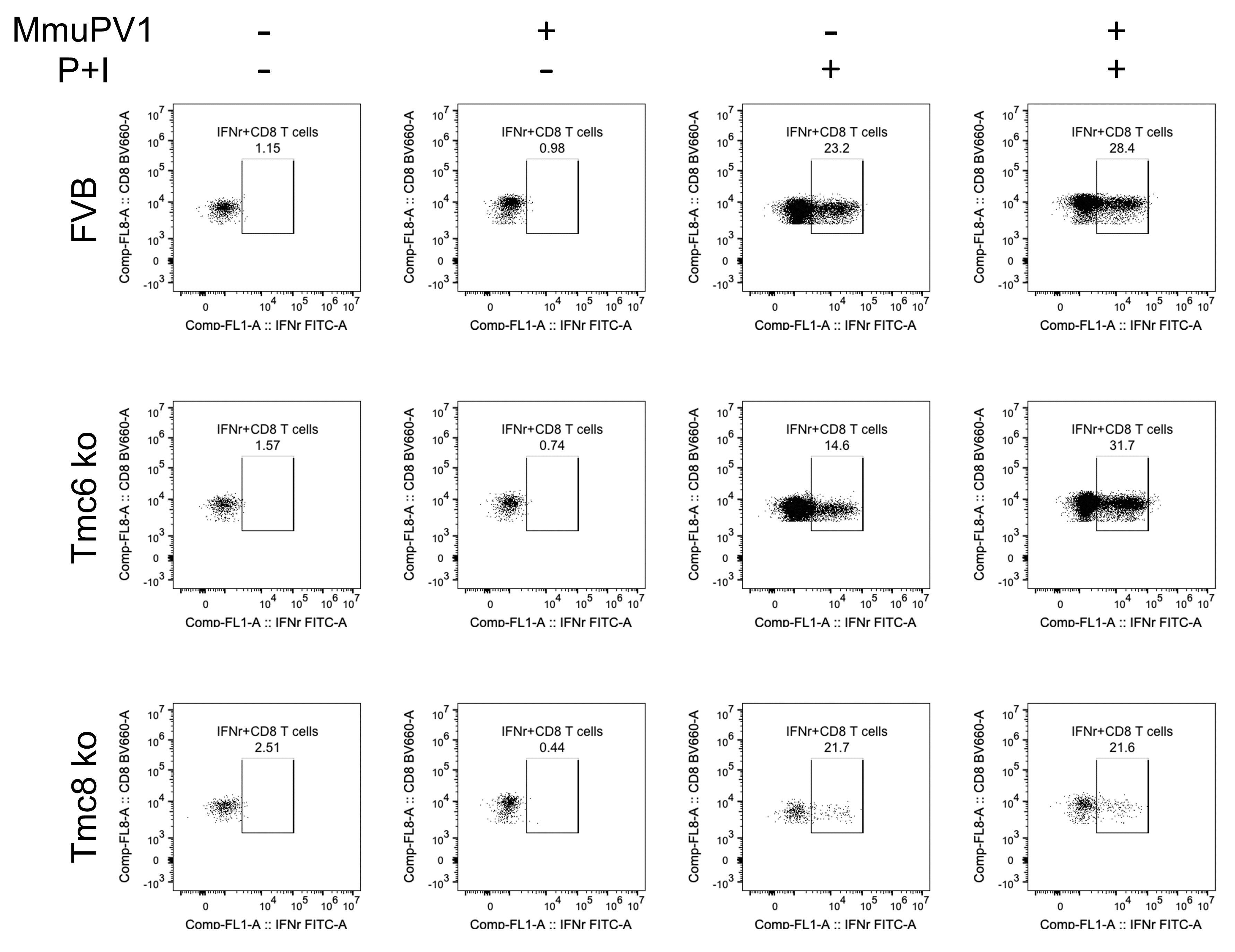

Supplement: S4 Fig — (TIF) [file ppat.1012837.s004.tif]

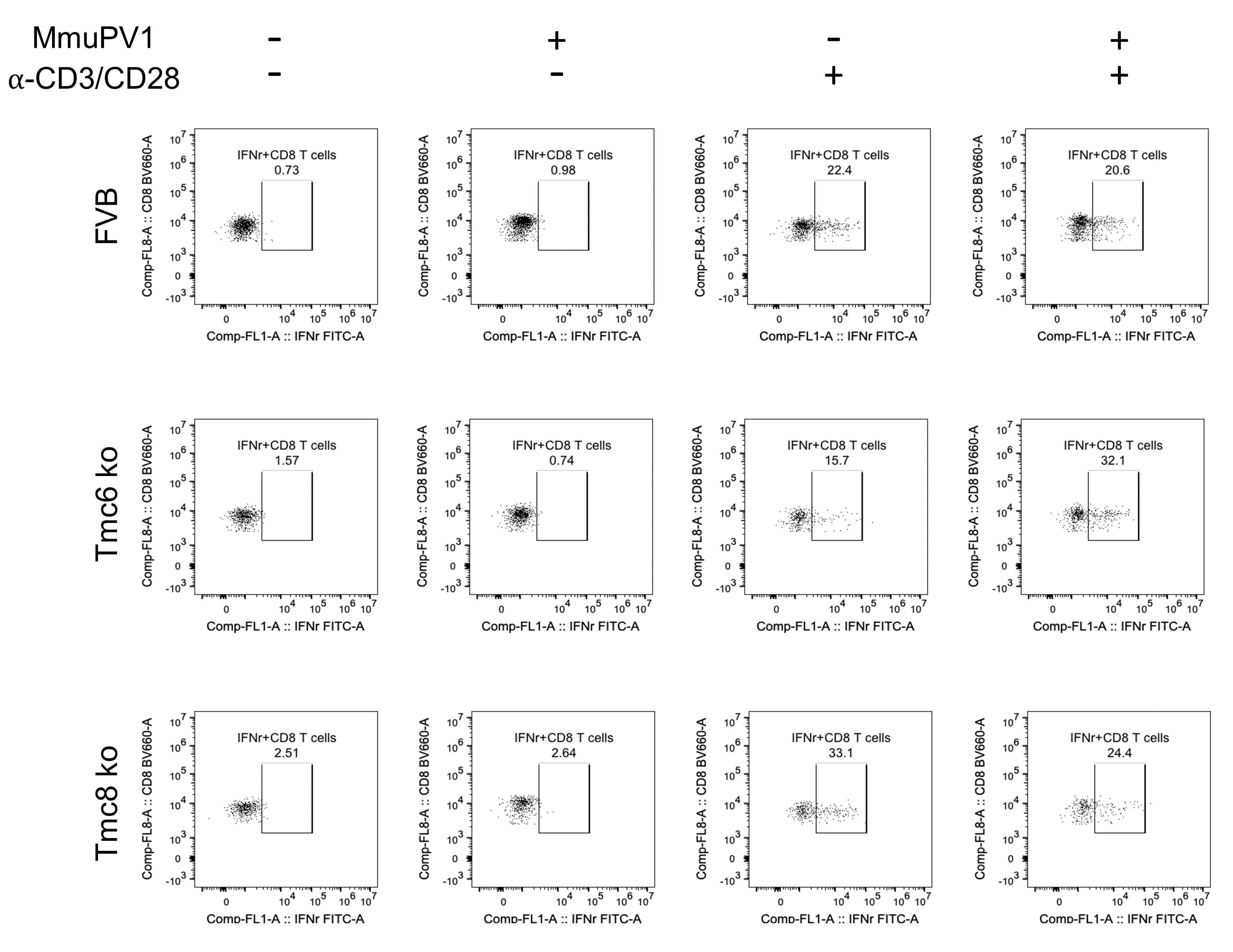

Supplement: S5 Fig — (TIF) [file ppat.1012837.s005.tif]

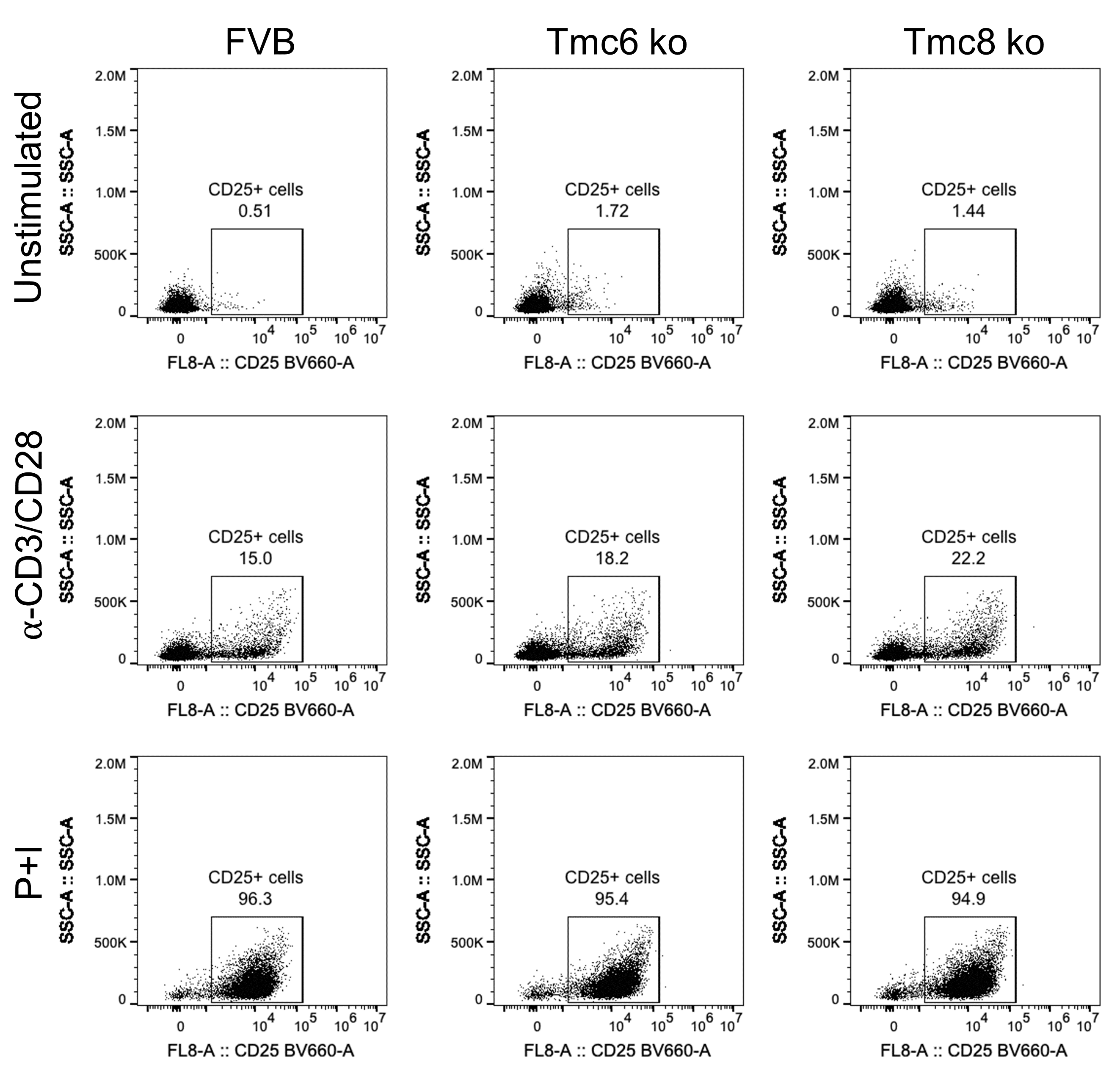

Supplement: S6 Fig — (TIF) [file ppat.1012837.s006.tif]

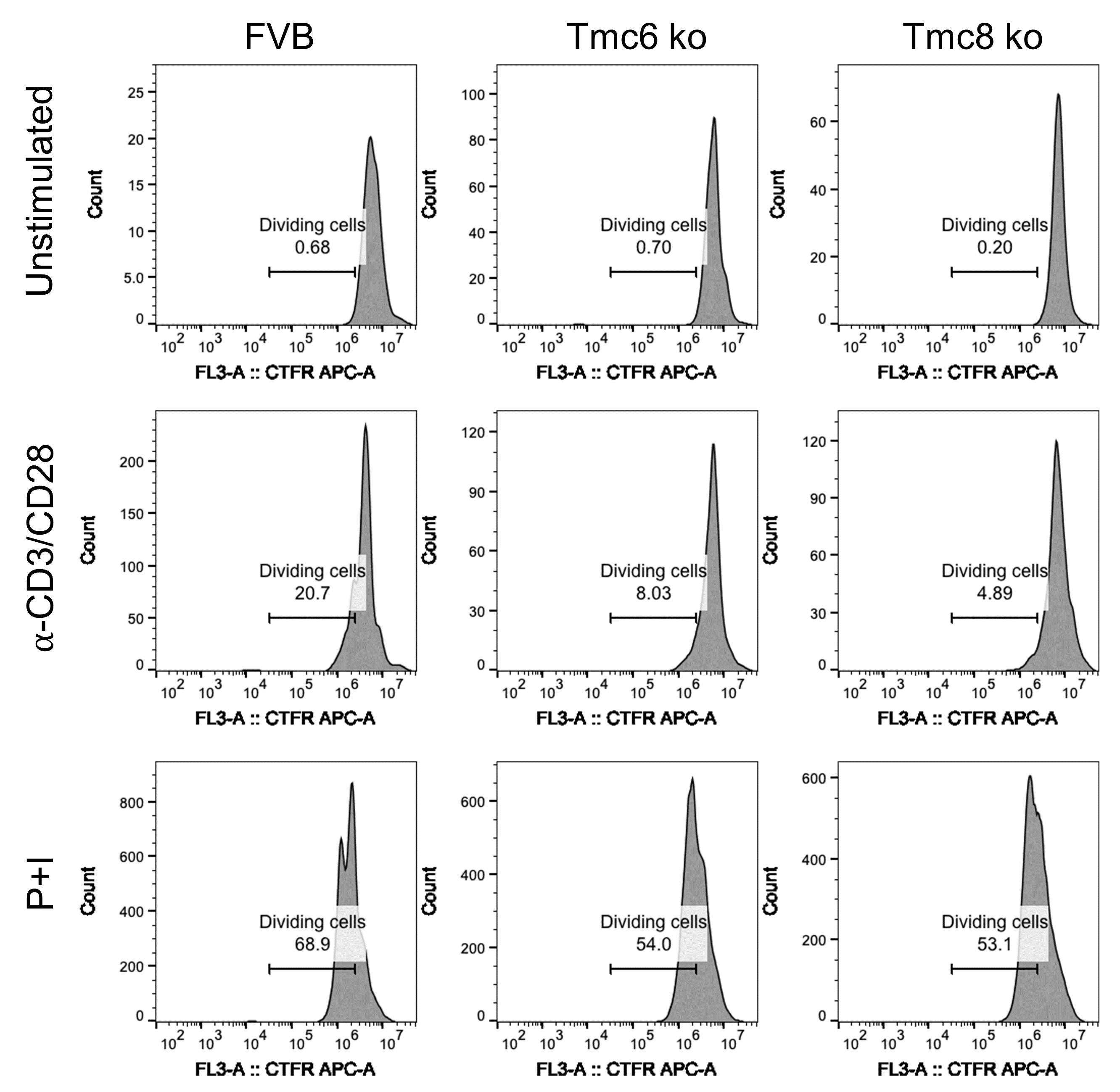

Supplement: S7 Fig — (TIF) [file ppat.1012837.s007.tif]

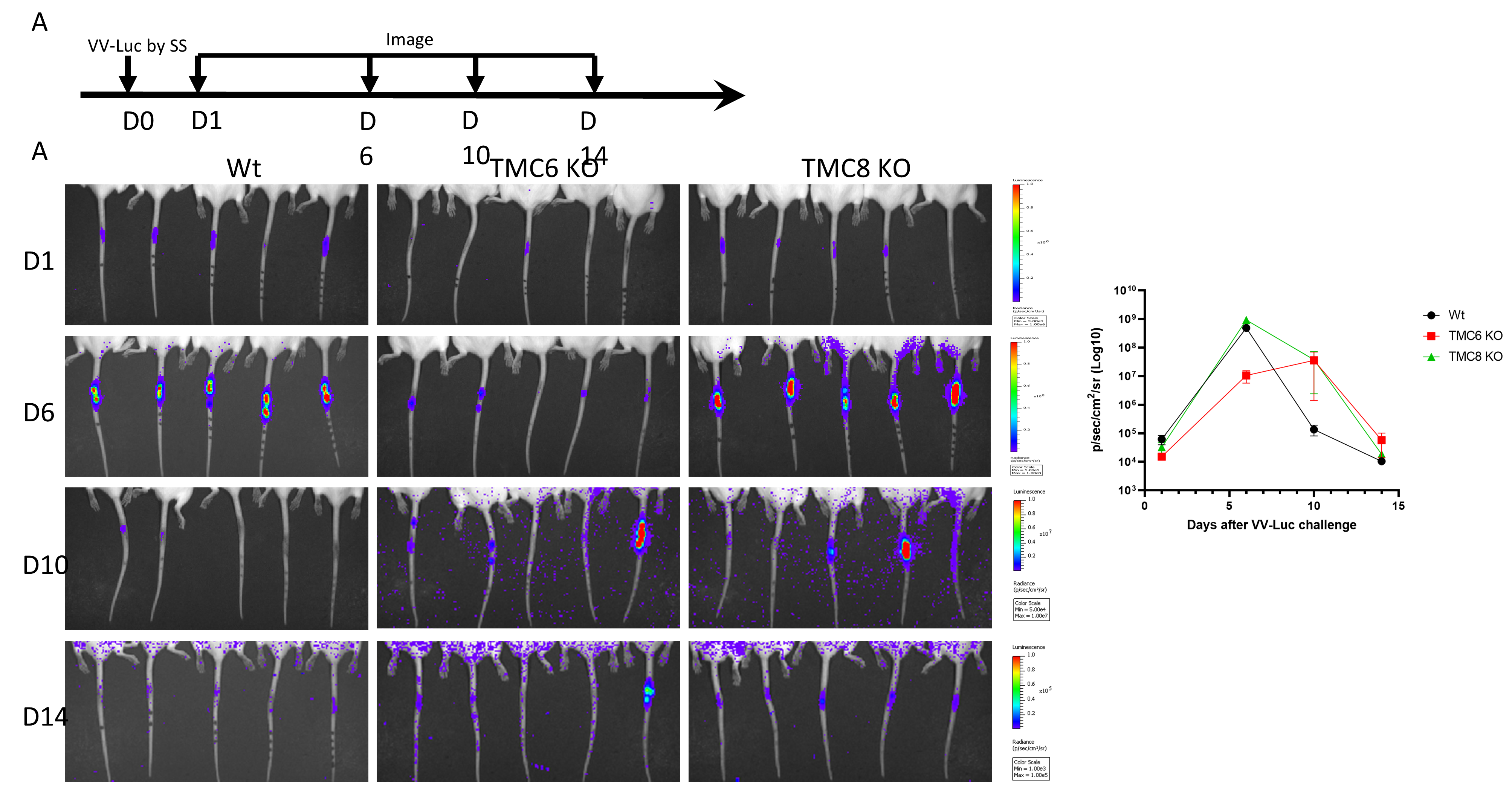

Supplement: S8 Fig — (A) Schematic illustration of the experiment. Briefly, 6–8 week old wild-type FVB, Tmc6-/-, or Tmc8-/- male mice (n = 5/group) anesthetized and 5x10^5^ pfu (5 μL) of vaccinia virus expressing luciferase was applied to tail skin, 1 cm from the base of the tail. The skin area was then gently scratched 15 times with a bifurcated needle. Mice were imaged by IVIS Spectrum in vivo imaging system series 2000 (PerkinElmer) at days 1, 6, 10 and 14. Total photon counts were quantified in the tumor site by using Living Image 2.50 software (PerkinElmer). (B) Representative luminescence imaging from each group. (C) Quantification of luminescence signal in interest area. (TIF) [file ppat.1012837.s008.tif]

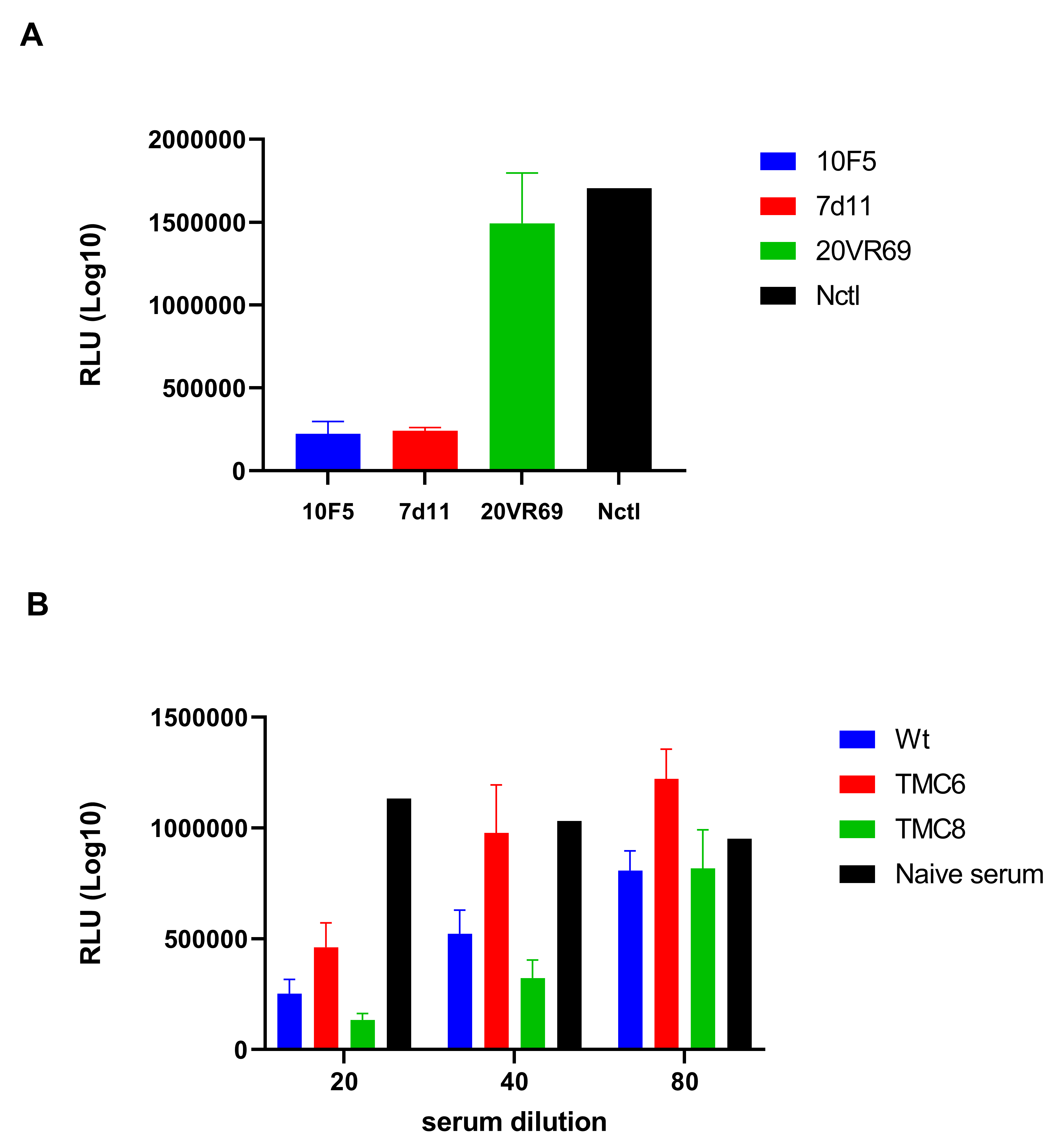

Supplement: S9 Fig — In vitro measurement of neutralizing serum antibody titer generated in response to percutaneous vaccinia virus challenge of wildtype, Tmc6-/- and Tmc8-/- FVB mice. (A) Anti-vaccinia virus monoclonal antibodies 10F5, 7d11 were used as positive control, and non-neutralizing serum 20VR69 as a negative control. (B) To assay neutralization titer of sera from challenged mice, 1x10^5^ pfu of vaccinia virus expressing luciferase (VV-luc) was pre-mixed with serially diluted sera for 1 h, then the mixture was added to 1 X 104 293TT cells in the well of a 96-well plate. After incubation for 24 h, cells were lysed, BPS Bioscience one step Luciferase Reagent buffer and Luciferase Reagent substrate were both added and luciferase activity measured in a luminometer. (TIF) [file ppat.1012837.s009.tif]

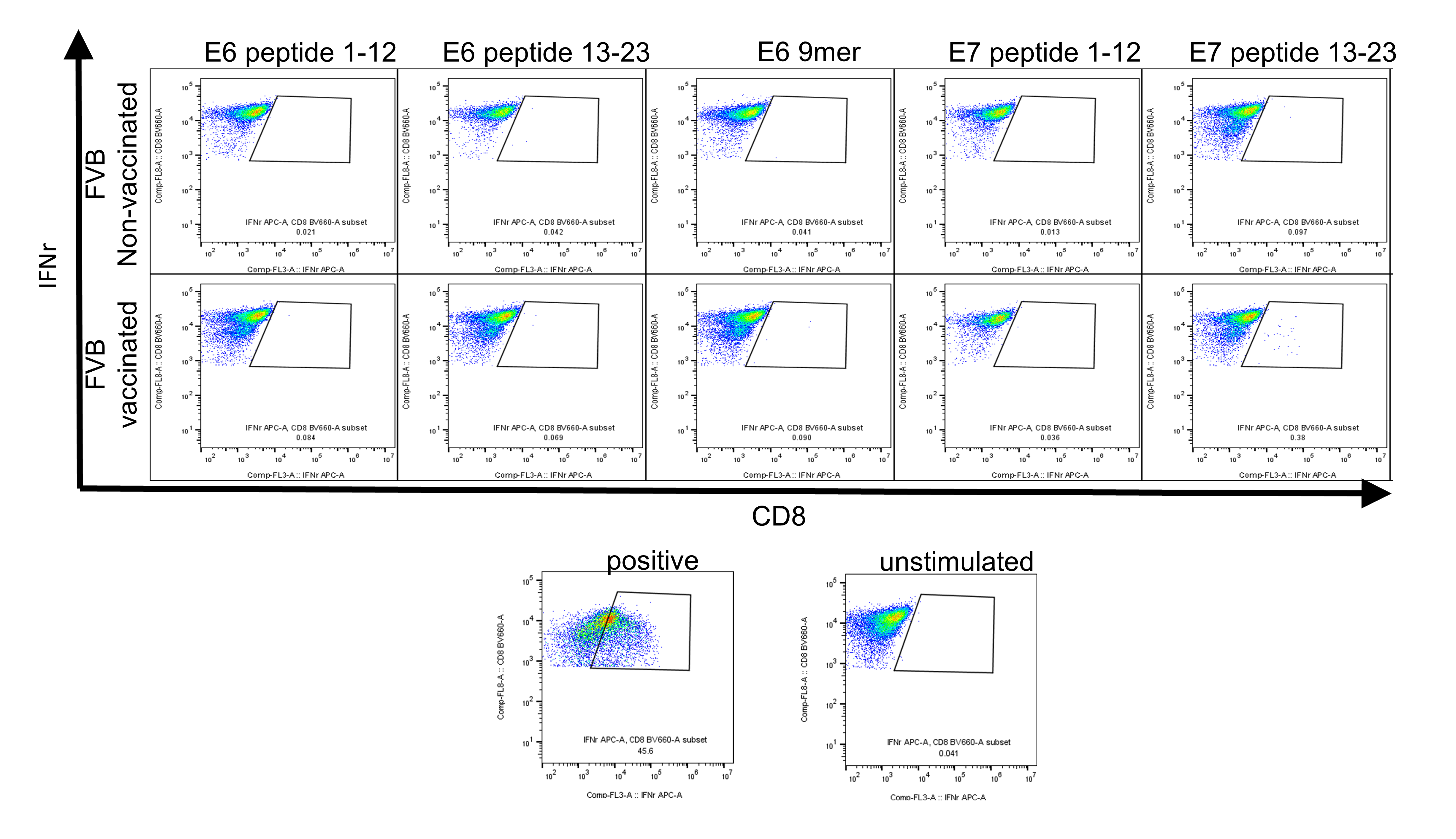

Supplement: S10 Fig — Characterization of MmuPV1 E6 and E7 epitopes recognized by CD8+ T cells of FVB mice. Mice (n = 3) were injected intramuscularly with 15μg of hCRT-mE6mE7mL2 DNA vaccine followed by electroporation on days 1, 8 and 15. One week after the final vaccination, splenocytes were collected, as were splenocytes from unvaccinated mice. To determine the epitopes, a panel of 20mer peptides, each overlapping by 15 amino acids were incubated with splenocytes in the presence of Golgi plug for 16 hours. Splenocytes stimulated with eBioscience Cell Stimulation Cocktail was used as a positive control. The cells were stained for interferon-γ and CD8, and analyzed by flow cytometry using a CytoFLEX S (Beckman) and data were analyzed by FlowJo software. Percentage of interferon-γ producing CD8+ T cells is presented. (TIF) [file ppat.1012837.s010.tif]

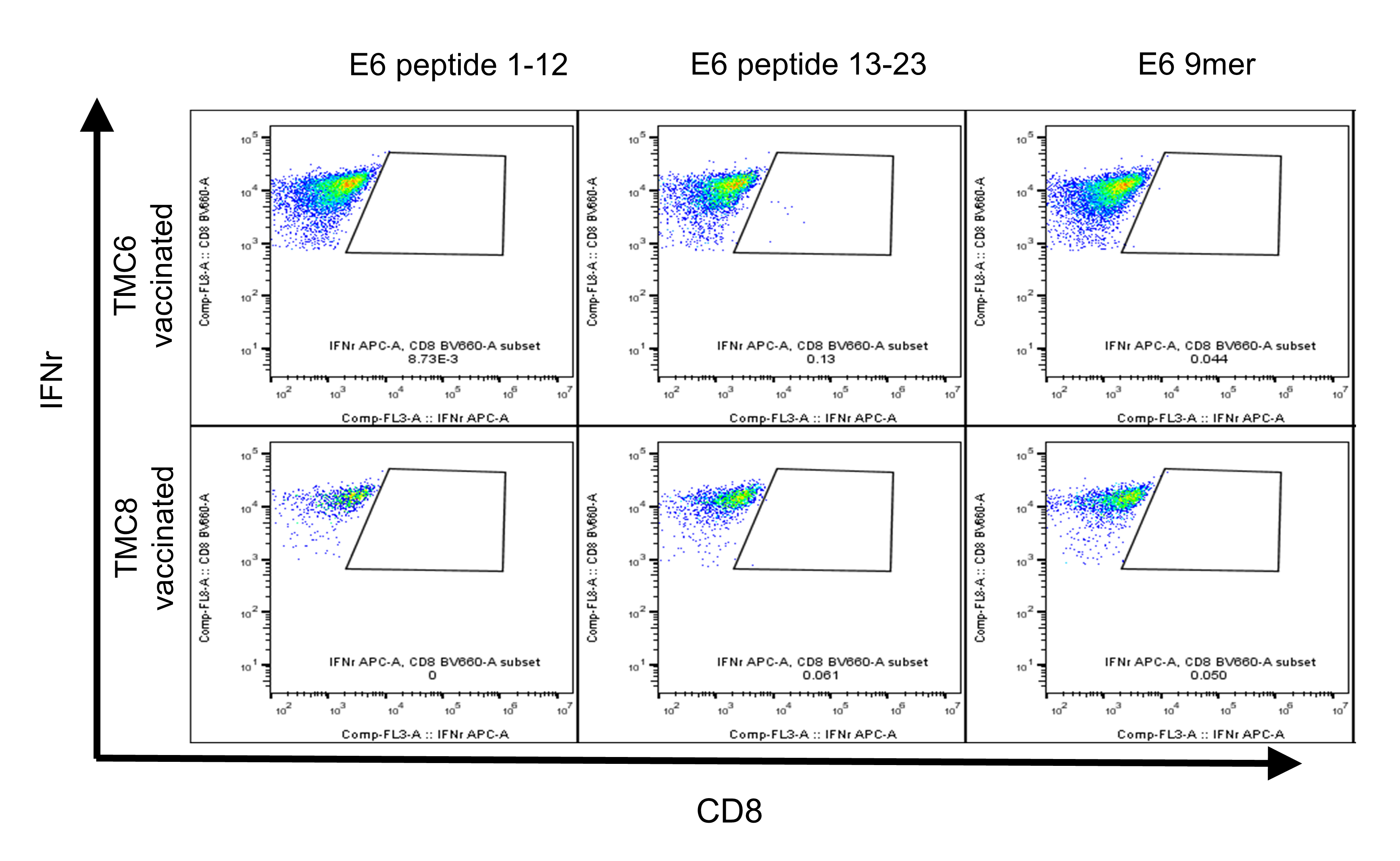

Supplement: S11 Fig — Characterization of MmuPV1 E6 epitopes recognized by CD8+ T cells of Tmc6-/- and Tmc8-/- FVB mice as in S10 Fig. (TIF) [file ppat.1012837.s011.tif]

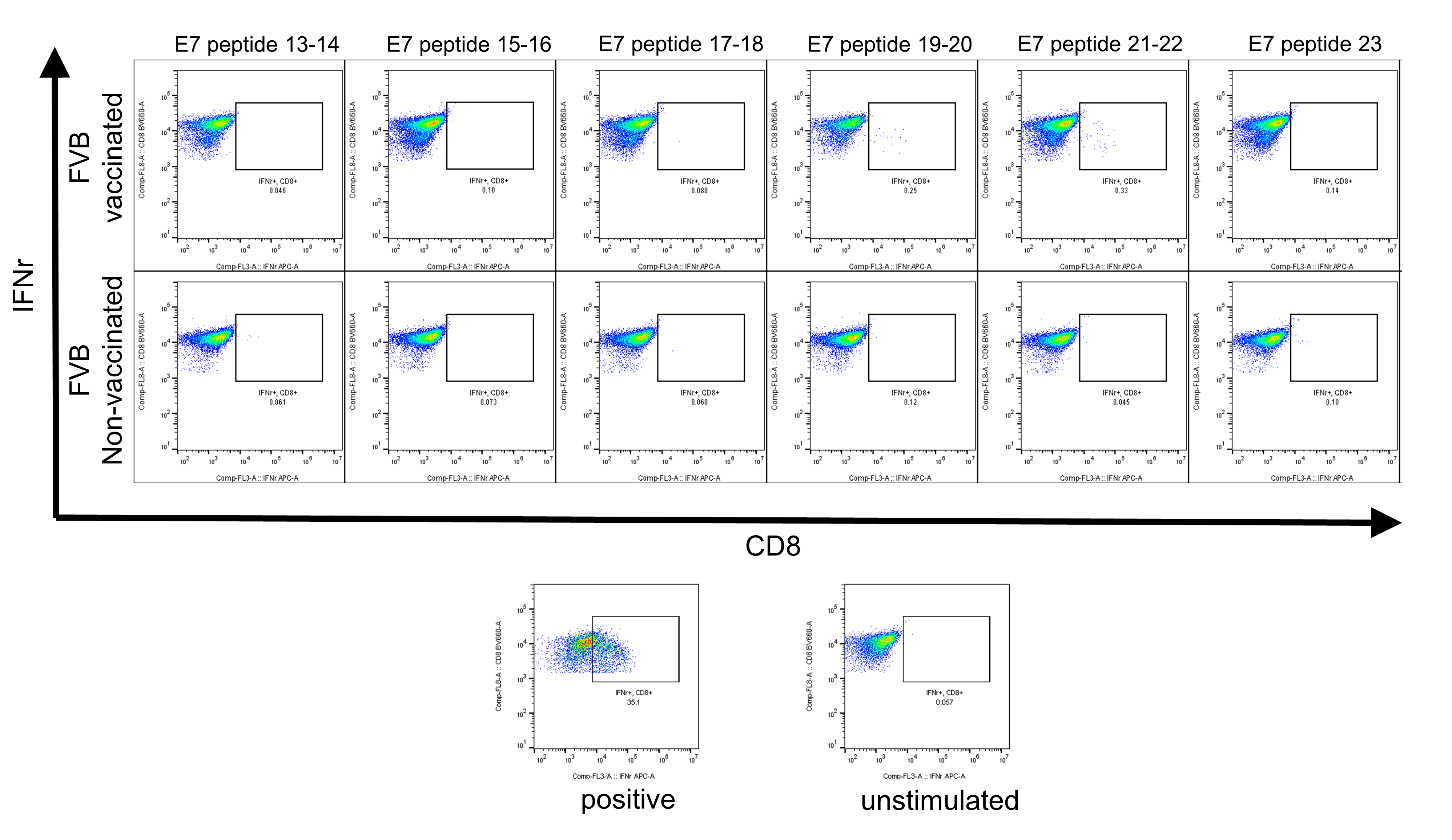

Supplement: S12 Fig — Characterization of MmuPV1 E7 epitopes recognized by CD8+ T cells of FVB mice as in S10 Fig. (TIF) [file ppat.1012837.s012.tif]

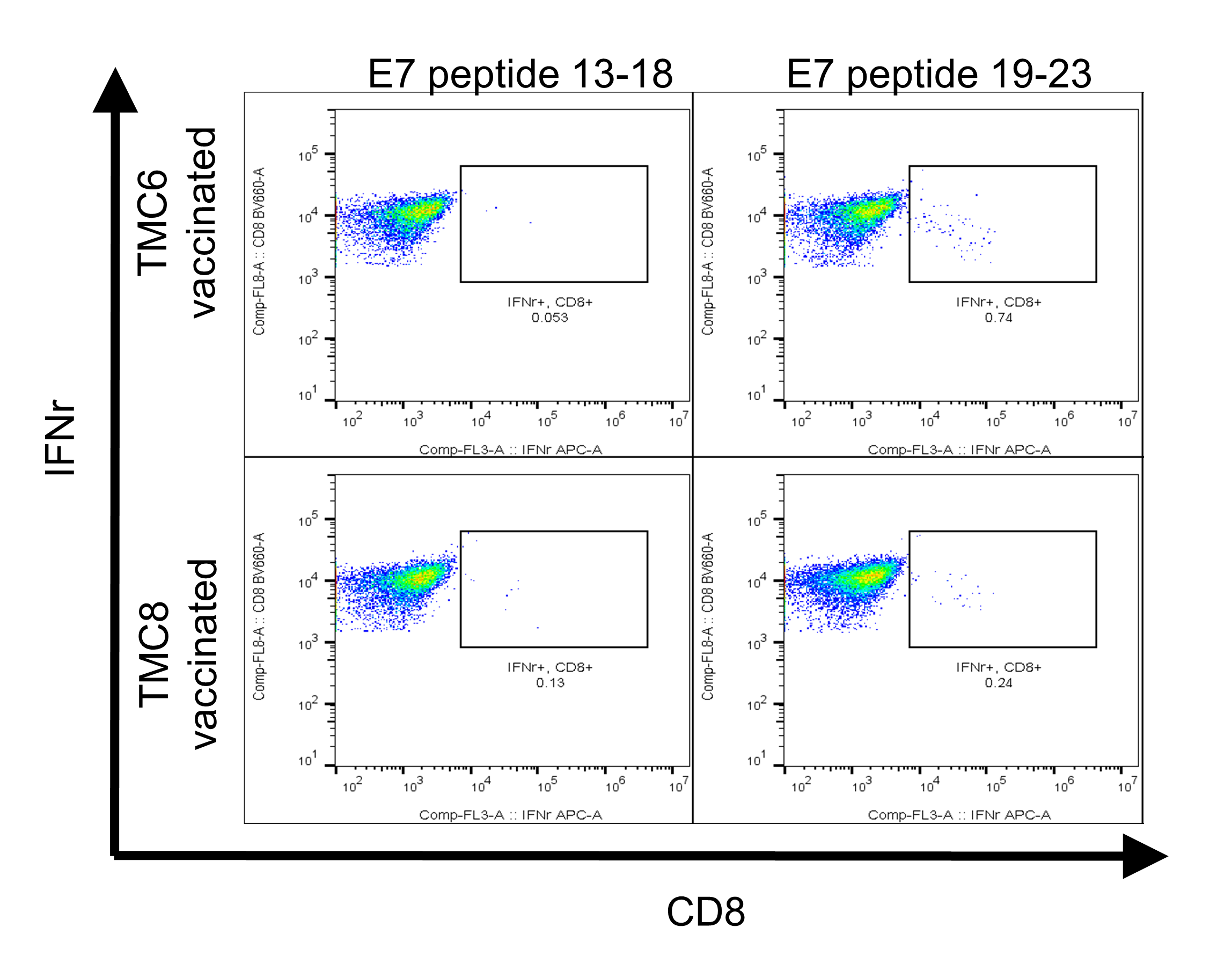

Supplement: S13 Fig — Characterization of MmuPV1 E7 epitopes recognized by CD8+ T cells of Tmc6-/- and Tmc8-/- FVB mice as in S10 Fig. (TIF) [file ppat.1012837.s013.tif]

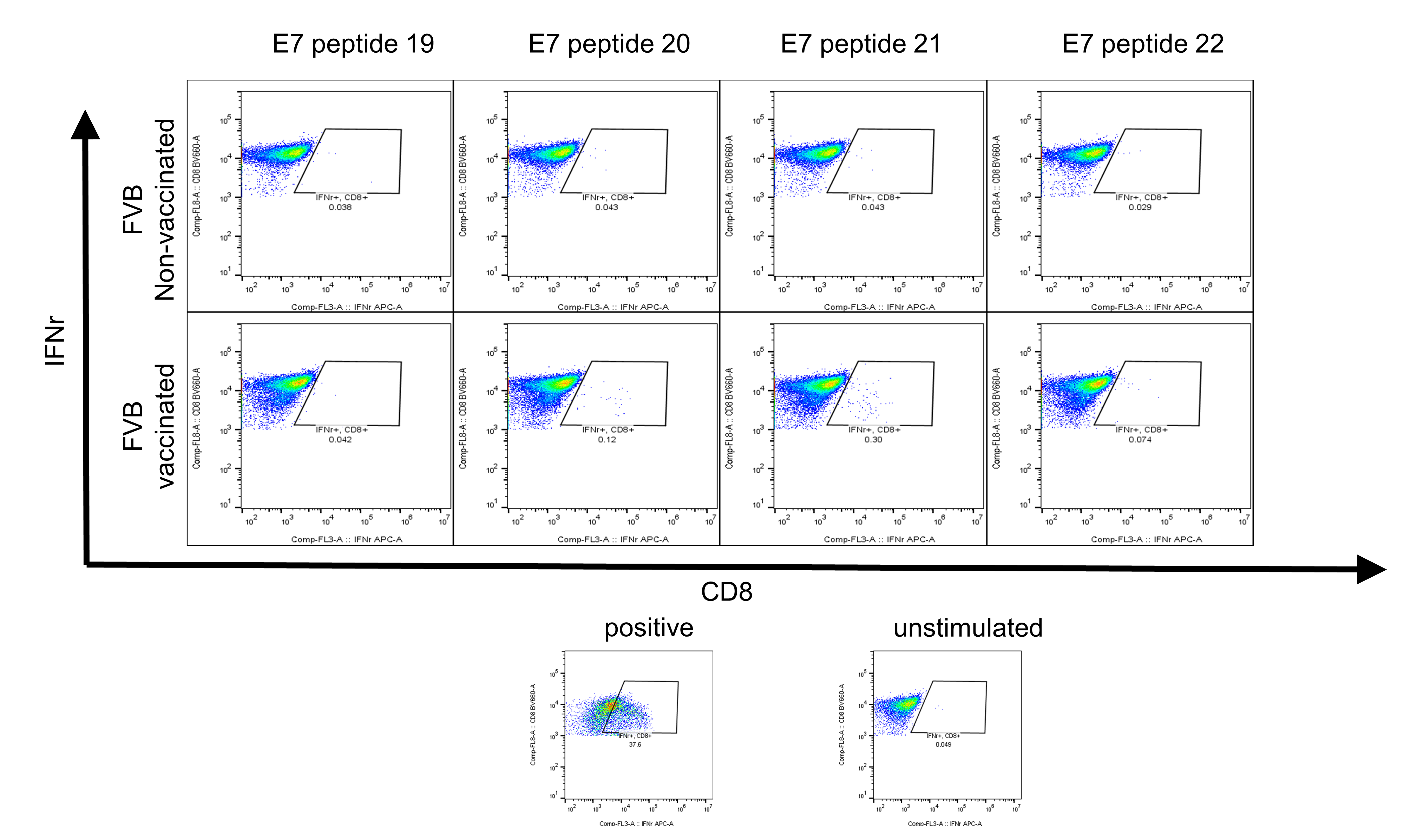

Supplement: S14 Fig — Characterization of MmuPV1 E7 epitopes recognized by CD8+ T cells of FVB mice as in S10 Fig. (TIF) [file ppat.1012837.s014.tif]

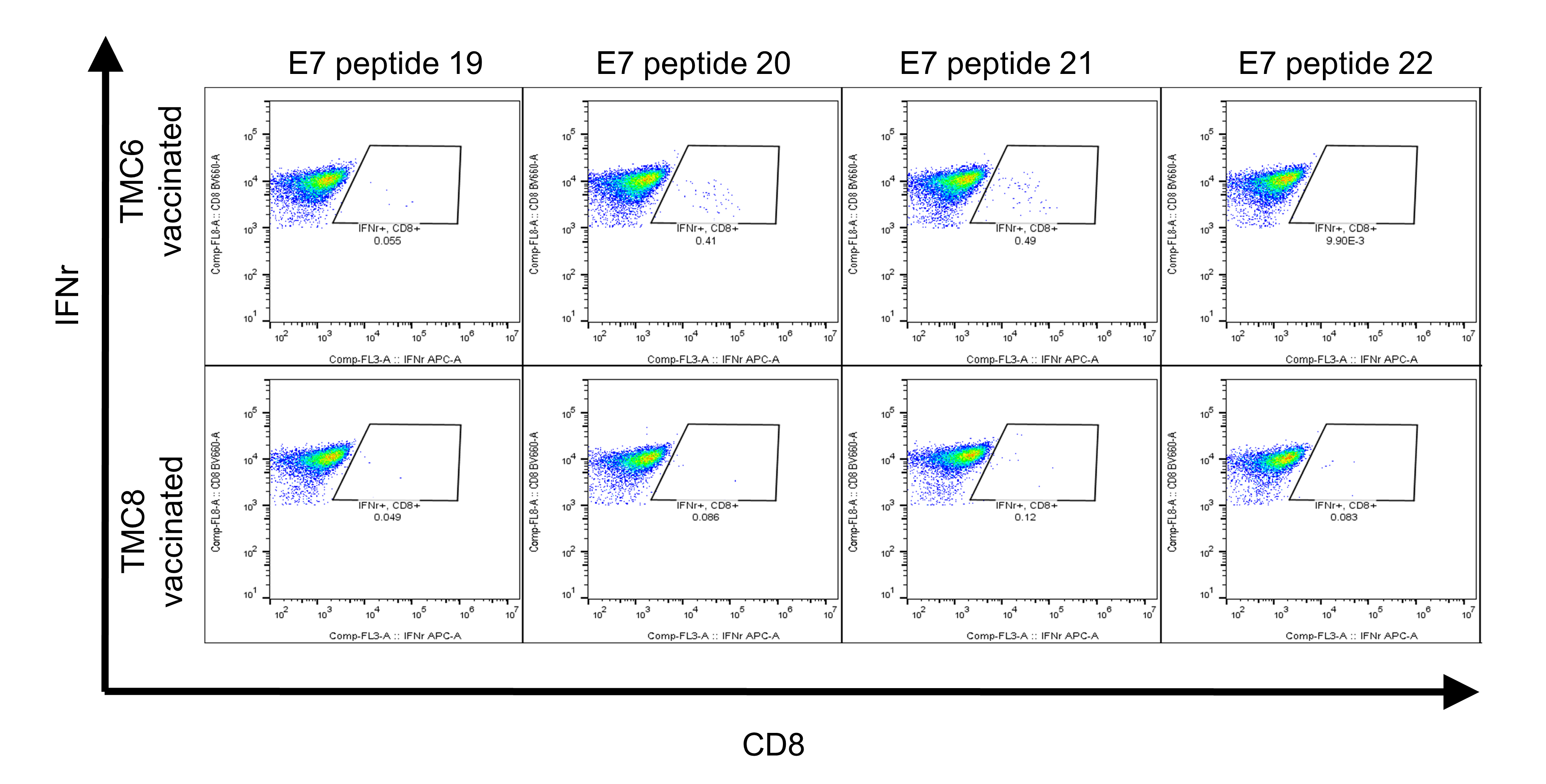

Supplement: S15 Fig — Characterization of MmuPV1 E7 epitopes recognized by CD8+ T cells of Tmc6-/- and Tmc8-/- FVB mice as in S10 Fig. (TIF) [file ppat.1012837.s015.tif]
