# Supplementary material for: MmuPV1 infection of Tmc6/Ever1 or Tmc8/Ever2 deficient FVB mice as a model of βHPV in typical epidermodysplasia verruciformis
Source: PLoS Pathog. 2025 Jan 15;21(1):e1012837. doi: 10.1371/journal.ppat.1012837 (PMC11734914; doi:10.1371/journal.ppat.1012837)
Supplement: S1 Table — A male mouse from each genotype, (A) wildtype FVB, (B) Tmc6-/-, or (C) Tmc8-/- of 2–3 months in age were sacrificed and keratinocytes cultured from the tails harvested. Keratinocytes were infected with 0.02 μL and 0.2 μL of MmuPV1 (1.86x10^9^ vge/μL), 3 wells each. The Cq for samples that did not amplify at 40 cycles were indicated as 40. (DOCX) [file ppat.1012837.s016.docx]

**Supplementary Table 1. MmuPV1 infection of keratinocytes cultured from *Tmc6*^-/-^, *Tmc8*^-/-^ or wild type mice.**

A male mouse from each genotype, (**A**) wild type FVB, (**B**) *Tmc6*^-/-^, or (**C**) *Tmc8*^-/-^ of 2-3 months in age were sacrificed and keratinocytes cultured from the tails harvested. Keratinocytes were infected with 0.02 µL and 0.2 µL of MmuPV1 (1.86x10^9^ vge/µL), 3 wells each. The Cq for samples that did not amplify at 40 cycles were indicated as 40.

**A**

| Genotype | MmuPV1 dose (µL) | MmuPV1 Cq | Capzb Cq | ∆Cq |
| --- | --- | --- | --- | --- |
| FVB | 0.2 | 29.08 | 19.96 | 9.12 |
|  | 0.2 | 30.24 | 19.76 | 10.48 |
|  | 0.2 | 29.25 | 20.34 | 8.91 |
|  | 0.02 | 30.06 | 21.73 | 8.33 |
|  | 0.02 | 28.63 | 20.21 | 8.42 |
|  | 0.02 | 32.53 | 21.12 | 11.41 |
|  | no virus | 40 | 20.45 | 19.55 |
|  | no virus | 40 | 20.4 | 19.60 |
|  | no virus | 40 | 19.93 | 20.07 |

**B**

| Genotype | MmuPV1 dose (µL) | MmuPV1 Cq | Capzb Cq | ∆Cq |
| --- | --- | --- | --- | --- |
| *Tmc6*-/- | 0.2 | 33.87 | 20.93 | 12.94 |
|  | 0.2 | 32.66 | 20.49 | 12.17 |
|  | 0.2 | 38.84 | 22.15 | 16.69 |
|  | 0.02 | 34.06 | 22.02 | 12.04 |
|  | 0.02 | 34.24 | 21.41 | 12.83 |
|  | 0.02 | 32.87 | 21.31 | 11.56 |
|  | no virus | 40 | 21.64 | 18.36 |
|  | no virus | 40 | 20.83 | 19.17 |
|  | no virus | 40 | 23.45 | 16.55 |

**C**

| Genotype | MmuPV1 dose (µL) | MmuPV1 Cq | Capzb  Cq | ∆Cq |
| --- | --- | --- | --- | --- |
| *Tmc8*-/- | 0.2 | 28.16 | 19.03 | 9.13 |
|  | 0.2 | 28.47 | 19.15 | 9.32 |
|  | 0.2 | 26.91 | 19.28 | 7.63 |
|  | 0.02 | 29.04 | 19.88 | 9.16 |
|  | 0.02 | 27.38 | 19.84 | 7.54 |
|  | 0.02 | 24.7 | 19.94 | 4.76 |
|  | no virus | 40 | 19.59 | 20.41 |
|  | no virus | 40 | 19.28 | 20.72 |
|  | no virus | 40 | 20.27 | 19.73 |
