# Supplementary material for: MmuPV1 infection of Tmc6/Ever1 or Tmc8/Ever2 deficient FVB mice as a model of βHPV in typical epidermodysplasia verruciformis
Source: PLoS Pathog. 2025 Jan 15;21(1):e1012837. doi: 10.1371/journal.ppat.1012837 (PMC11734914; doi:10.1371/journal.ppat.1012837)
Supplement: S2 Table — The Cq for samples that did not amplify at 40 cycles were indicated as 40. (DOCX) [file ppat.1012837.s017.docx]

**Supplementary Table 2. RT-PCR analysis of MmuPV1 and *Capzb* transcript levels at 2 months after tail challenge.**

The Cq for samples that did not amplify at 40 cycles were indicated as 40.

| Genotype | MmuPV1  Cq | Capzb  Cq | ΔCq |
| --- | --- | --- | --- |
| FVB | 40 | 21.51 | 18.49 |
| FVB | 40 | 21.05 | 18.95 |
| FVB | 40 | 22.05 | 17.95 |
| FVB | 40 | 22.01 | 17.99 |
| FVB | 40 | 21.63 | 18.37 |
| *Tmc6-/-* | 40 | 22.34 | 17.66 |
| *Tmc6-/-* | 39.40 | 21.46 | 17.94 |
| *Tmc6-/-* | 37.51 | 21.03 | 16.48 |
| *Tmc6-/-* | 40 | 21.06 | 18.94 |
| *Tmc6-/-* | 38.46 | 21.21 | 17.25 |
| *Tmc8-/-* | 40 | 21.28 | 18.72 |
| *Tmc8-/-* | 40 | 21.29 | 18.71 |
| *Tmc8-/-* | 40 | 21.44 | 18.56 |
| *Tmc8-/-* | 40 | 21.23 | 18.77 |
| *Tmc8-/-* | 32.21 | 21.40 | 10.81 |
| nude | 15.01 | 23.11 | -8.10 |
